# Development of a D-xylose fermenting and inhibitor tolerant industrial *Saccharomyces cerevisiae* strain with high performance in lignocellulose hydrolysates using metabolic and evolutionary engineering

**DOI:** 10.1186/1754-6834-6-89

**Published:** 2013-06-21

**Authors:** Mekonnen M Demeke, Heiko Dietz, Yingying Li, María R Foulquié-Moreno, Sarma Mutturi, Sylvie Deprez, Tom Den Abt, Beatriz M Bonini, Gunnar Liden, Françoise Dumortier, Alex Verplaetse, Eckhard Boles, Johan M Thevelein

**Affiliations:** 1 Laboratory of Molecular Cell Biology, Institute of Botany and Microbiology, KU Leuven, Belgium; 2 Department of Molecular Microbiology, VIB, Kasteelpark Arenberg 31, B-3001 Leuven, Heverlee, Flanders, Belgium; 3 Institute of Molecular Biosciences, Goethe-University Frankfurt, Max-von-Laue-Str. 9, D-60438 Frankfurt am Main, Germany; 4 Department of Chemical Engineering, Lund University, P.O. Box 124, 22100 Lund, Sweden; 5 Laboratory of Enzyme, Fermentation and Brewing Technology, KAHO Sint-Lieven University College, KU Leuven Association, Gebroeders De Smetstraat 1, 9000, Ghent, Flanders, Belgium

**Keywords:** Bioethanol, Lignocellulose, D-xylose fermentation, D-xylose isomerase, Inhibitor tolerance, *Saccharomyces cerevisiae*, Evolutionary engineering

## Abstract

**Background:**

The production of bioethanol from lignocellulose hydrolysates requires a robust, D-xylose-fermenting and inhibitor-tolerant microorganism as catalyst. The purpose of the present work was to develop such a strain from a prime industrial yeast strain, Ethanol Red, used for bioethanol production.

**Results:**

An expression cassette containing 13 genes including *Clostridium phytofermentans XylA*, encoding D-xylose isomerase (XI), and enzymes of the pentose phosphate pathway was inserted in two copies in the genome of Ethanol Red. Subsequent EMS mutagenesis, genome shuffling and selection in D-xylose-enriched lignocellulose hydrolysate, followed by multiple rounds of evolutionary engineering in complex medium with D-xylose, gradually established efficient D-xylose fermentation. The best-performing strain, GS1.11-26, showed a maximum specific D-xylose consumption rate of 1.1 g/g DW/h in synthetic medium, with complete attenuation of 35 g/L D-xylose in about 17 h. In separate hydrolysis and fermentation of lignocellulose hydrolysates of *Arundo donax* (giant reed), spruce and a wheat straw/hay mixture, the maximum specific D-xylose consumption rate was 0.36, 0.23 and 1.1 g/g DW inoculum/h, and the final ethanol titer was 4.2, 3.9 and 5.8% (v/v), respectively. In simultaneous saccharification and fermentation of Arundo hydrolysate, GS1.11-26 produced 32% more ethanol than the parent strain Ethanol Red, due to efficient D-xylose utilization. The high D-xylose fermentation capacity was stable after extended growth in glucose. Cell extracts of strain GS1.11-26 displayed 17-fold higher XI activity compared to the parent strain, but overexpression of XI alone was not enough to establish D-xylose fermentation. The high D-xylose consumption rate was due to synergistic interaction between the high XI activity and one or more mutations in the genome. The GS1.11-26 had a partial respiratory defect causing a reduced aerobic growth rate.

**Conclusions:**

An industrial yeast strain for bioethanol production with lignocellulose hydrolysates has been developed in the genetic background of a strain widely used for commercial bioethanol production. The strain uses glucose and D-xylose with high consumption rates and partial cofermentation in various lignocellulose hydrolysates with very high ethanol yield. The GS1.11-26 strain shows highly promising potential for further development of an all-round robust yeast strain for efficient fermentation of various lignocellulose hydrolysates.

## Background

The yeast *Saccharomyces cerevisiae* is still the dominant organism for industrial bioethanol production owing to its high rate of fermentation of hexose sugars, high tolerance to ethanol, inhibitors, acidity and other industrial process conditions, well-established production, storage and transport systems at commercial scale, comprehensive physiological and molecular knowledge and its genetic tractability [1,2]. Unfortunately, baker’s yeast is unable to efficiently metabolize pentose sugars, particularly D-xylose, which accounts for up to 35% of total sugars in xylan-rich lignocellulosic biomass such as hard woods and straw [3]. Although there are various species of bacteria, filamentous fungi and other yeast species that are naturally capable of efficiently metabolizing D-xylose, they lack the other crucial advantages of the yeast *S. cerevisiae*, which have made it the most prominent industrial microorganism. Lignocellulose hydrolysates contain various inhibitors depending on the type of biomass and pretreatment methodology used, making extreme inhibitor tolerance a crucial trait for reaching economically viable second-generation bioethanol production [4,5]. The inherently higher robustness and tolerance of *S. cerevisiae* to various inhibitors gives it a head start in programs aimed at developing strains with extreme inhibitor tolerance, able to efficiently ferment hexoses and pentoses in concentrated lignocellulose hydrolysates [6]. Although progress has been made in developing strains with higher ethanol and inhibitor tolerance in bacteria, like *Escherichia coli*, and in other yeast species, like *Scheffersomyces (Pichia) stipitis*, these strains still lag far behind industrial *S. cerevisiae* strains in their level of ethanol tolerance, general robustness and performance under industrial conditions [7,8].

The engineering of novel metabolic capacities into robust microorganisms may be easier than the alternative strategy, i.e. engineering of very high ethanol tolerance and prominent general robustness. Impressive progress has been made in engineering pentose fermentation capacity into the yeast *S. cerevisiae*[9,10]. For that purpose, two heterologous pathways for D-xylose utilization have been utilized. First, the genes encoding D-xylose reductase (XR) and xylitol dehydrogenase (XDH) from *Scheffersomyces (Pichia) stipitis* have been expressed in *S. cerevisiae*. This resulted in D-xylose fermentation, but also in significant production of xylitol under anaerobic conditions, which is due to NADH/NADPH cofactor imbalance of XR and XDH [11]. The performance of these strains has been improved considerably by addressing the cofactor imbalance and by over-expression of endogenous xylulokinase (XK) and enzymes of the non-oxidative part of the pentose phosphate pathway [12-17].

The second pathway allows direct isomerization of D-xylose to xylulose through heterologous expression of xylose isomerase (XI). After the first successful attempt to express the thermophilic bacterium *Thermus thermophilus* XI into *S. cerevisiae *[18], recombinant strains expressing the fungal *Piromyces sp.* strain E2 xylose isomerase have been reported with better enzymatic activity [19,20]. By using an isomerization instead of a reduction/oxidation conversion of D-xylose to xylulose, the problem of co-factor imbalance is avoided. However, the rate of D-xylose utilization in XI expressing strains was found to be inferior to that in strains harboring the XR/XDH pathway [21]. This was mostly attributed to the low activity of the XI enzyme in *S. cerevisiae* and its inhibition by xylitol, generated from reduction of D-xylose by the endogenous enzymes encoded by *GRE3,* GCY1, YPR1, YDL124W and YJR096W [22-24]. The level of xylitol produced is much lower, however, than in the strains expressing the XR/XDH pathway. Deletion of *GRE3* in an XI expressing strain improved both the rate of D-xylose consumption and ethanol production [25]. The aldose reductase, encoded by *GRE3,* plays a role in stress protection and its deletion is therefore not desirable in industrial yeast strains [26]. To overcome these problems, Brat *et al., *[27] constructed the first recombinant *S. cerevisiae* strain demonstrating high activity of prokaryotic XI, using codon-optimized *XylA* gene from *Clostridium phytofermentans.* This enzyme was much less inhibited by xylitol compared to the enzyme from *Piromyces*. Nevertheless, the rate of D-xylose consumption and ethanol production by this recombinant strain was still slow.

Different metabolic and evolutionary engineering strategies have been used successfully to improve D-xylose utilization in a yeast strain expressing *Piromyces* xylose isomerase. Overexpression of genes encoding xylulokinase and enzymes of the non-oxidative part of the pentose phosphate pathway, combined with deletion of *GRE3* to reduce xylitol formation, considerably improved the D-xylose consumption rate [20]. This finally resulted in strains with strong pentose fermentation capacity and partial cofermentation of glucose and D-xylose [28,29]. Moreover, the xylose isomerase pathway was compatible with the bacterial L-arabinose utilization pathway, in contrast to the XR/XDH pathway [30]. These results suggested that the xylose isomerase pathway might be the pathway of choice for constructing superior industrial yeast strains with optimal fermentation performance in lignocellulose hydrolysates [31]. However, all these engineered strains were still made in a haploid laboratory yeast strain background, displaying in general suboptimal fermentation performance and poor robustness and stress tolerance, which makes them unsuitable for use in industrial fermentations. Since previous work showed that XI expressing strains displayed higher yield of ethanol per consumed D-xylose compared to strains harboring the XR/XDH pathway [21] and since they profit from direct isomerization of D-xylose to xylulose without cofactor requirement, the XI pathway seemed to be most promising to engineer into a robust industrial yeast strain.

In this work, we have selected Ethanol Red as industrial host strain to engineer high-capacity pentose-fermentation, because it is one of the most widely used yeast strains for first-generation bioethanol production. The strain has excellent fermentation capacity, high robustness and stress tolerance, and also displays excellent performance in fed-batch production on molasses, is tolerant to dehydration and retains high vitality during storage and transport. Using this strain, we have developed the first industrial *S. cerevisiae* strain that converts D-xylose to ethanol with a yield close to the theoretical maximum yield and with a very high specific rate of fermentation. For that purpose, a recombinant strain was first constructed by chromosomal integration of codon-optimized *XylA* from *C. phytofermentans* in an over-expression gene cassette containing genes of the non-oxidative pentose phosphate pathway and the D-xylose transporting hexose transporter *HXT7*. Subsequently, we have used Ethyl Methanesulfonate (EMS) mutagenesis, genome shuffling and selection in lignocellulose hydrolysate, enriched with D-xylose, and subsequent evolutionary adaptation in complex medium with D-xylose, to greatly enhance both D-xylose utilization efficiency and inhibitor tolerance. The activity of XI was dramatically increased in the evolved strain, but other genetic changes were also required for its superior D-xylose fermentation capacity in lignocellulose hydrolysates.

## Results

### Insertion of D-xylose utilization cassette into the Ethanol Red strain

**Figure 1:**
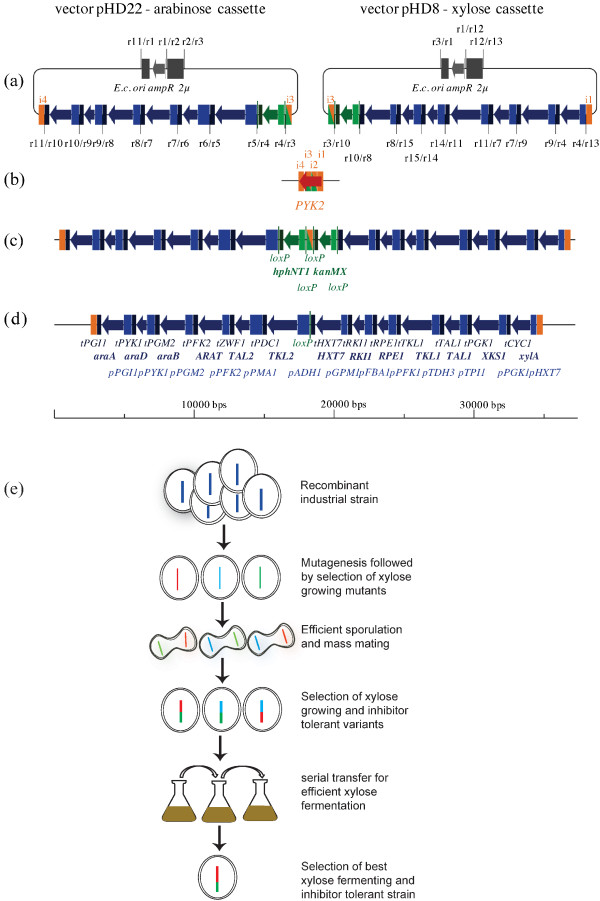
**General strategy used for development of a strain with high xylose fermentation capacity and high inhibitor tolerance.** (**a**) to (**d**): Scheme of the designed plasmids and the integration of the cassettes into the *pyk2* allele of the Ethanol Red strain. (**a**) Vectors carrying the expression cassettes for xylose and arabinose utilization; (**b**) scheme of wild type *PYK2* locus; (**c**) scheme of *pyk2* locus with integrated arabinose- and xylose-utilization cassettes; (**d**) locus after loxP-Cre mediated recombination displaying the genotype of the strain HDY.GUF5. Non-integratable parts of the plasmids are colored in gray, parts where the homologous recombination took place in orange (numbered i1 - i4), the integrative cassette in blue, and the green color symbolizes parts of the cassette which can pop out by loxP-Cre mdiated recombination. Restriction sites: r1 - StuI; r2 - NheI; r3 - SbfI; r4 - Bsu36I; r5 - NarI; r6 - AvrII; r7 - AscI; r8 - BamHI; r9 - NotI; r10 - PmeI; r11 - XmaI; r12 - AatII; r13 - BstZ17I; r14 - SapI; r15 - PshAI. (**e**) Evolutionary engineering strategy. The diploid recombinant industrial strain was mutagenized and mutant strains able to grow on xylose were selected. Their genome was shuffled by sporulation and mass mating, and the best D-xylose utilizers selected in D-xylose-enriched pre-treated spruce hydrolysate. The culture was then submitted to evolutionary adaptation in YP + 40 g/L D-xylose, single clones were evaluated in different stages and the strain with the best performance selected from the last stage.

We have constructed and stably integrated D-xylose- and L-arabinose-utilization gene expression cassettes into both alleles of the *pyk2* locus in the diploid industrial bioethanol production strain Ethanol Red (Fermentis, a division of S. I. Lesaffre, Lille, http://www.fermentis.com/) (Figure 1a to d). The *PYK2* gene was chosen as it encodes a dispensable glucose-repressed second isoform of pyruvate kinase, which is only expressed on non-fermentable carbon sources [32]. The integrative gene expression cassettes were designed to minimize the number of transformations and the remaining *loxP* scars after removal of the selection markers. Within the cassettes, 13 heterologous and endogenous genes involved in pentose utilization were put under the control of constitutive and strong yeast promoters. Some of the genes were codon-optimized according to the highly efficient glycolytic codon usage of yeast [33]. The first cassette contains genes coding for codon-optimized xylose isomerase (XI) from *Clostridium phytofermentans*[27]*,* codon-optimized yeast xylulokinase (Xks1), the yeast pentose/hexose transporter Hxt7 [34] and all the enzymes of the non-oxidative part of the pentose phosphate pathway: transketolase (Tkl1), transaldolase (Tal1), ribulose-5-phosphate 3-epimerase (Rpe1) and ribose-5-phosphate ketol-isomerase (Rki1). The second cassette includes codon-optimized genes for the arabinose transporter (AraT) from *Scheffersomyces (Pichia) stipitis*[35], codon-optimized arabinose isomerase (AraA) from *Bacillus licheniformis*, codon-optimized ribulokinase (AraB) and ribulose-5-p 4-epimerase (AraD) from *E. coli*[33] as well as codon-optimized yeast transketolase 2 (Tkl2) and transaldolase 2 (Nqm1) (Figure 1a). Both cassettes were stably integrated into both *PYK2* alleles, followed by removal of the antibiotic resistance markers, resulting in strain HDY.GUF5. Despite the presence of two copies of both gene cassettes, the strain hardly fermented any D-xylose or L-arabinose to ethanol. For the further improvement of this strain, we focused first on D-xylose fermentation performance, mainly because D-xylose is the dominant pentose sugar in lignocellulose hydrolysates, compared to the minor amounts of L-arabinose usually present [3]. In addition, it was unclear whether L-arabinose utilization capacity might compromise D-xylose utilization capacity in this strain background, since previous data showed loss of D-xylose fermentation ability by a recombinant D-xylose utilizing strain after evolutionary adaptation for improved L-arabinose utilization [36]. Therefore, the recombinant strain HDY.GUF5 was subjected to several consecutive methodologies for strain improvement: mutagenesis, genome shuffling and selection, followed by multiple rounds of evolutionary adaptation for D-xylose fermentation (Figure 1e).

### EMS mutagenesis

**Figure 2:**
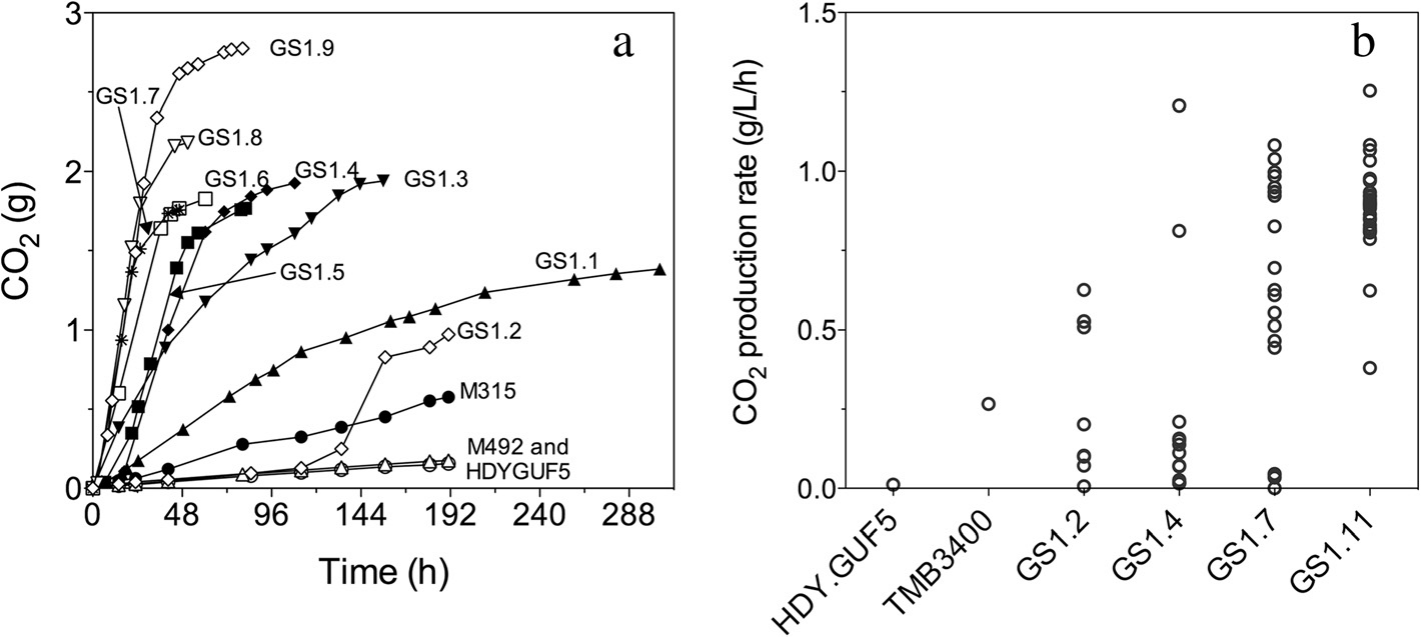
**Gradual establishment of efficient D-xylose utilization during the mutagenesis and multiple evolutionary engineering steps.** (**a**) CO_2_ production as measured by weight loss in sequential semi-anaerobic batch fermentations in YP + 40 g/L xylose at 35°C. After EMS mutagenesis and one step of genome shuffling, the culture was submitted to 11 serial transfers, in which each time part of the culture after the batch fermentation was used to start a new fermentation. The CO_2_ production profile of the first 9 serial batch fermentations is shown. The concentration of xylose was increased to 50 g/L and 60 g/L in the 8th and 9th batch fermentation, respectively. (GS1 stands for the first step of genome shuffling, and the next number indicates the step in the serial transfer.) (**b**) Volumetric CO_2_ production in semi-anaerobic batch fermentation in YP + 40 g/L xylose at 35°C by single cell isolates obtained from the 2nd (GS1.2), 4th (GS1.4), 7th (GS1.7) and 11th (GS1.11) serial batch fermentation during the evolutionary adaptation process. The horizontal bar represents the mean with the standard deviation. The parent strain, HDY.GUF5, and a previously constructed industrial D-xylose utilizing stain, TMB3400, [37] are shown for comparison.

The diploid HDY.GUF5 strain was treated with 3% EMS for 0.5, 1, 2, 3, and 4 h in order to create diverse populations of mutant strains with possibly beneficial mutations for D-xylose utilization. The mutagenized cells were plated out onto YPX plates (with 20 g/L D-xylose) and also on YPD plates (with 20 g/L glucose) to estimate the cells‘ survival rate. After incubation for 72 h at 35°C, 4 colonies from the 4 h treated and 3 colonies from the 3 h treated cells grew on YPX plates. For the EMS untreated cells and those treated for 0.5 to 2 h, no colonies were observed on YPX plates. The seven D-xylose-growing strains were plated for single colonies and further tested in semi-anaerobic batch fermentations with D-xylose. The rate of D-xylose fermentation by all mutants was extremely slow except for mutant M315, which showed slightly better fermentation compared to the parent strain HDY.GUF5 (Figure 2a). M315 also displayed twice faster growth rate (0.037 h^-1^ ± 0.004) compared to the parent strain (0.019 h^-1^ ± 0.0004) in synthetic medium containing D-xylose as sole carbon source.

### Genome shuffling

The sporulation efficiency of the seven mutant strains was evaluated prior to the genome shuffling step. Only one of the mutant strains, M492, was still able to sporulate. The 3 to 4 h EMS treatment possibly caused mutations abolishing sporulation capacity in the other strains. The best D-xylose utilizing mutant strain M315 had ***MAT****α* mating type and was shown to be diploid by flow cytometry, indicating that the strain was a ***MAT****α/α* diploid. The M315 mutant was able to mate with ***MATa ***cells and we then used the two mutant strains, M315 and M492, together with the parent strain HDY.GUF5 for the genome shuffling step. The parent strain was included to facilitate loss of deleterious mutations.

The M492 mutant strain and the parent strain HDY.GUF5 were sporulated to more than 75% efficiency and the spores were liberated by zymolyase treatment. The mass of isolated spores from the two strains was allowed to germinate in YPD and then mass-mated with exponentially growing cells of the mutant M315. The zygotes from the mass mating were subsequently allowed to proliferate at 35°C in synthetic medium containing D-xylose as sole carbon source. The OD_600_ increased from 2.5 to 12 in 24 h. The whole cell population was then transferred into undetoxified spruce hydrolysate, supplemented with YP and 40 g/L D-xylose. The concentration of spruce hydrolysate used prevented growth of the parent strain HDY.GUF5, but the shuffled culture was able to grow in 48 h from an initial OD_600_ of 2 to an OD_600_ of 26. Acid pre-treated spruce hydrolysate supplemented with 40 g/L D-xylose was chosen for selection, because it contains a high amount of inhibitors and only a limited amount of D-glucose (13 g/L). When the glucose was used up, the strains continued to grow on the supplemented D-xylose, allowing us to select inhibitor tolerant mutants without losing the capacity to grow on D-xylose.

Eventually, the isolated spores from M492 and HDY.GUF5 were evaluated individually for spore viability by spreading the isolated spores on YPD plates. While none of the spores from M492 tested were viable, the expected number of cells (about 10^3^) were germinated from the parent HDY.GUF5, indicating that mainly the HDY.GUF5 and the mutant M315 were involved in the genome shuffling step, while the M492 strain likely had a much lower or no contribution at all.

### Directed evolution

In order to enrich for fast D-xylose utilizing clones and subsequently improve the D-xylose utilization rate, the entire population of cells obtained after genome shuffling and subsequent selection in spruce hydrolysate with D-xylose, was used for the evolutionary engineering process. The cells were first grown aerobically in shake flasks containing 40 ml YPX medium for 48 h and then used for inoculation at an initial OD_600_ of 2.75 into cylindrical 150 ml fermentation tubes containing 100 ml YP medium with 40 g/L D-xylose. The fermentations were performed at 35°C under semi-anaerobic conditions, which were attained by slow stirring of the culture at 120 rpm to insure mixing of the cells without significant aeration. This method gradually created semi-anaerobic conditions (oxygen level of less than 1 ppm) within 1 h of incubation. The fermentation rate of the first culture, called GS1.1 was slow but already much better compared to the original strains used in the genome shuffling (Figure 2a). We then performed ten additional serial transfers using the same medium, each time with an initial cell density with OD_600_ of 5 (equivalent to 1.3 g DW/L). A relatively high inoculation density was used to insure that new variants of the cell population that were generated during the evolutionary process were effectively transferred to the next batch. In addition, complex medium, rather than defined mineral medium, was chosen for cultivation to avoid selective pressure due to nutrient limitation. As a result, the D-xylose utilization rate was the main selective criterion.

In the second culture, GS1.2, the lag phase was much longer than in the first culture, GS1.1, probably due to loss of viability during the prolonged incubation of the GS1.1 culture. However, a sharp rise in D-xylose consumption rate, as indicated by the CO_2_ production rate, was observed after 112 h (Figure 2a). To avoid possible loss of viability because of substrate depletion, subsequent serial transfers were performed each time before complete D-xylose depletion. Considerable improvement in the rate of D-xylose consumption was observed with each round of evolutionary adaptation (Figure 2a). The most dramatic change happened in the 3rd culture, GS1.3, in which almost no lag phase was observed, as opposed to the 112 h lag phase in the previous GS1.2 culture. In the 8th, 9th and 10th culture, the concentration of D-xylose was increased to 50 g/L, 60 g/L and 100 g/L, respectively, to further adapt the yeast to higher D-xylose concentrations with the assumption that higher concentrations of D-xylose might increase the rate of fermentation due to higher flux through the pathway. For the last culture, GS1.11, 40 g/L D-xylose was used again to make sure that the strain could also utilize lower D-xylose concentrations at a similar rate.

The progress of the evolutionary engineering process was continuously monitored by evaluation of single cell clones. A total of 9, 15, 20 and 27 single cell isolates from the 2nd, 4th, 7th and 11th culture, respectively, that were able to grow well on YPX plates, were evaluated for fermentation performance in YP + 40 g/L D-xylose (Figure 2b). A previously constructed industrial D-xylose utilizing stain, TMB3400, expressing xylose reductase and xylitol dehydrogenase [37], and the parent strain HDY.GUF5, were included for comparison (Figure 2b). Some isolates from the 2nd and 4th culture were already better than TMB3400 in terms of D-xylose fermentation rate (Figure 2b). However, all isolates from the 7th and 11th culture showed a much faster rate and much higher extent of D-xylose utilization than TMB3400 (Figure 2b). The individual clones isolated from the 7th and 11th culture showed a similar rate and extent of fermentation and therefore the evolutionary adaptation process was terminated after the 11th culture.

### Selection of the best D-xylose fermenting strain

**Figure 3:**
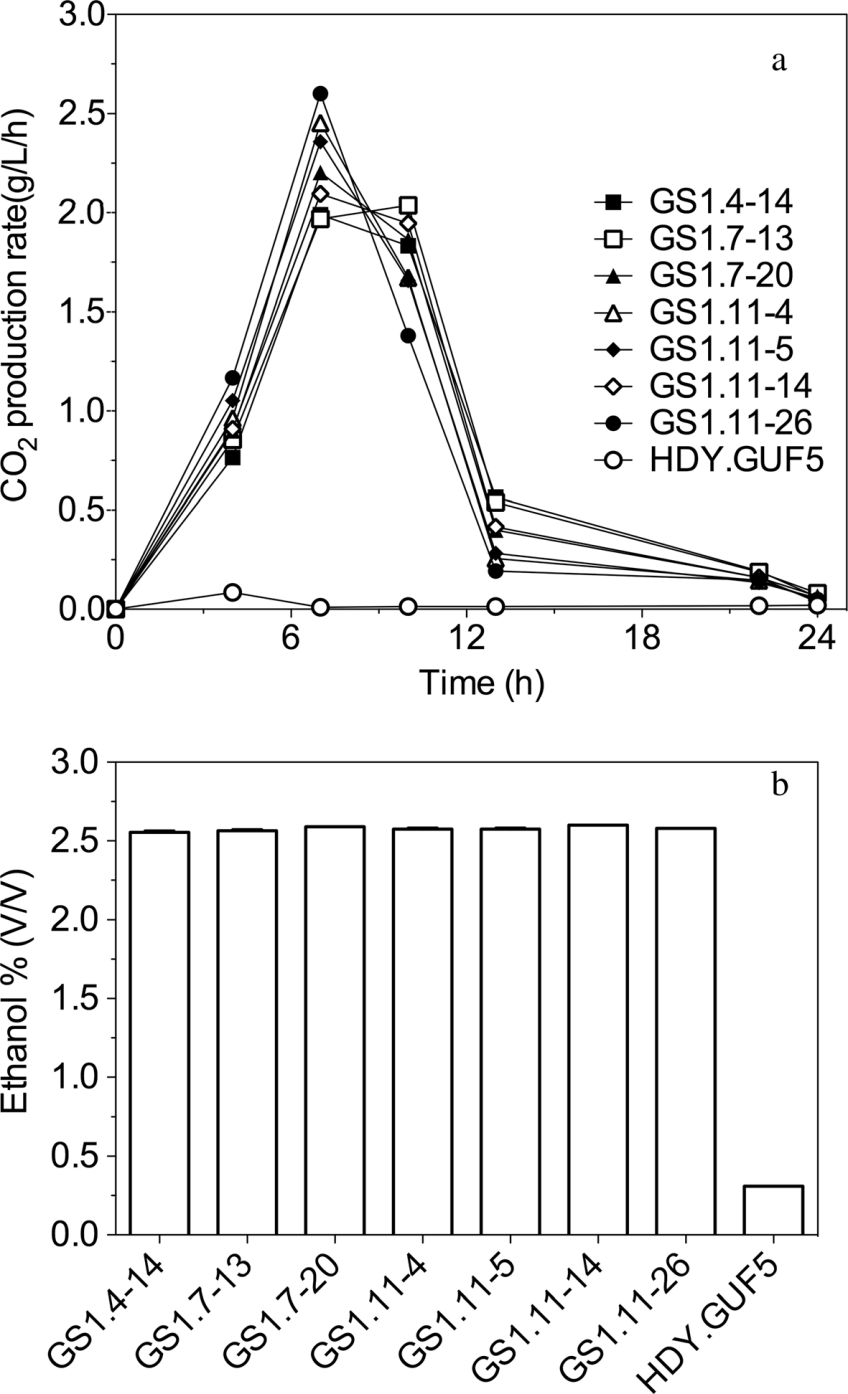
**D-xylose fermentation by superior single-cell isolates from different serial batch fermentations in the evolutionary engineering procedure.** (**a**) CO_2_ production as measured by weight loss in semi-anaerobic batch fermentations in YP + 40 g/L xylose at 35°C. Selected single cell isolates from each of the 4th (GS1.4), 7th (GS1.7) and 11th (GS1.11) culture were used. The parent strain, HDY.GUF5, was used for comparison. (**b**) Final ethanol titer reached in the fermentations of (**a**). Each experiment was performed in duplicate, and error bars represent standard deviation from the average of duplicate values.

Seven of the best individual clones from the 4th, 7th and 11th culture were tested in more detail for fermentation performance and ethanol yield in YP medium with 40 g/L D-xylose. The course of fermentation with the best strains from the cultures GS1.4, GS1.7 and GS1.11, as well as the control strain HDY.GUF5, is shown in Figure 3a. The final ethanol level for these fermentations is shown in Figure 3b. The seven isolates showed a similar performance with a slight difference in the rate of CO_2_ production and final ethanol yield. Ethanol yield of up to 0.48 g/g was obtained for most of the strains, with 40 g/L D-xylose as the main carbon source and an initial cell density of 1.3 g DW/L. This corresponds to 94% of the theoretical maximal ethanol production. There was almost no xylitol and little glycerol produced. Although the final ethanol level reached by these strains was very similar, the isolate GS1.11-26 reproducibly showed the highest rate of fermentation (Figure 3a) and was selected for further characterization.

**Figure 4:**
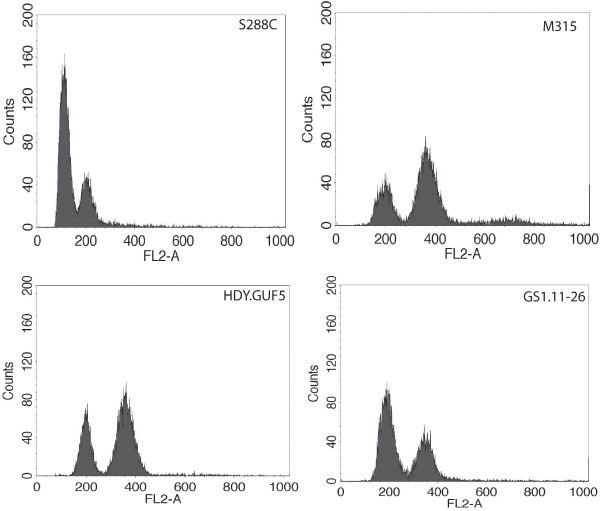
**Comparison of DNA content among parent and mutant strains**, **as determined by flow cytometry.** DNA content is shown for a haploid control strain S288c, mutant M315, diploid parent strain HDY.GUF5 and strain GS1.11-26, the best performing single cell isolate obtained from the 11th culture. Mutant M315 and GS1.11-26 appear to be diploid.

We also tested mating type and ploidy of the best performing single cell clones from the 11th culture, GS1.11. The GS1.11-26 strain and all other strains tested, as well as the mutant M315, were found to be ***MAT****α* and to have a diploid DNA content (Figure 4 and results not shown). We also performed a pheromone assay for mating type to rule out the possibility that the mating type PCR failed due to SNPs in the HMR locus, which might cause failure to detect ***MATa***. However, the pheromone assay confirmed that the strains were ***MAT****α* (results not shown). Hence, we can conclude that the best D-xylose utilizing clones were ***MAT****α/α* diploids*.*

### Fermentation performance of strain GS1.11-26 with D-xylose and a glucose/D-xylose mixture

**Figure 5:**
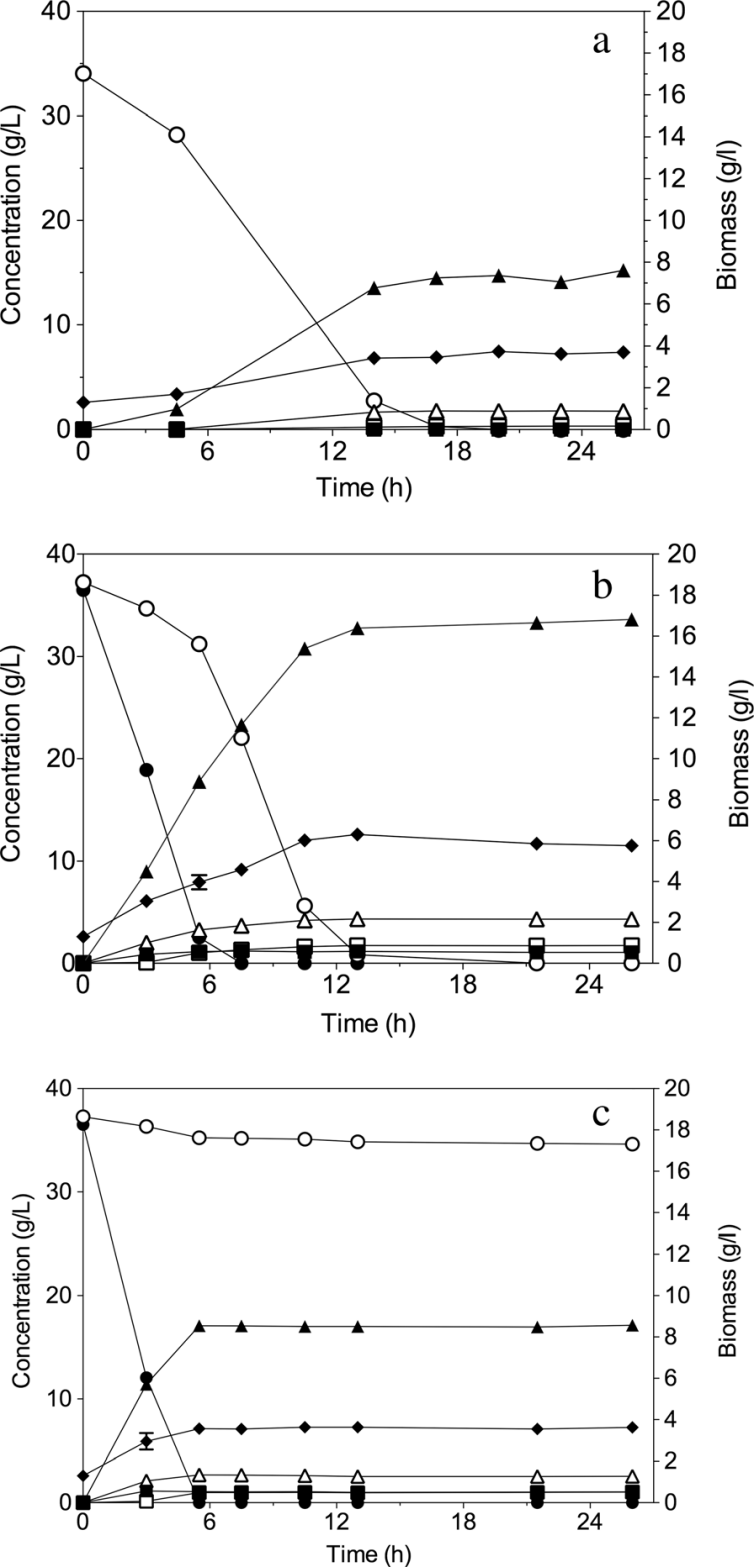
**Performance of strain GS1.11-26 in semi-anaerobic batch fermentations with D-xylose and a glucose/D-xylose mixture.** (**a**) Semi-anaerobic batch fermentation with strain GS1.11-26 in synthetic medium with D-xylose. (**b**) Semi-anaerobic batch fermentation in rich YP medium containing 36 g/L glucose and 37 g/L D-xylose with strain GS1.11-26. (**c**) Semi-anaerobic batch fermentation in rich YP medium containing 36 g/L glucose and 37 g/L D-xylose with parent strain HDY.GUF5. (●) Glucose, (○) D-xylose, (▲) ethanol, (∆) glycerol, (■) acetate, (□) D-xylitol and (⁬) biomass. Each experiment was performed in duplicate, and error bars represent standard deviation from the average of duplicate values.

The fermentation performance of the strain GS1.11-26 was evaluated in semi-anaerobic batch fermentation at 35°C with an initial cell density of 1.3 g DW/L. Synthetic medium with D-xylose and YP medium with a mixture of D-xylose and glucose was used (Figure 5). An air-tight fermentation lock containing glycerol was used to avoid entrance of air. Samples were taken every few h with needles.

In synthetic medium with 35 g/L D-xylose as sole carbon source, the evolved strain consumed all the available D-xylose in about 17 h, (Figure 5a) with maximum D-xylose consumption rate of 1.10 g/g DW/h and maximum ethanol production rate of 0.48 g/g DW/h. The final yield of ethanol was 0.46 g/g D-xylose and the xylitol yield was less than 0.01 g/g D-xylose. Though yeast strains expressing XI produce less xylitol compared to those of XR/XDH expressing strains [19], the presence of other enzymes such as the non-specific aldose reductase encoded by *GRE3* results in conversion of D-xylose to xylitol [38]. Since the xylitol yield in the strain GS1.11-26 was very low, we sequenced the *GRE3* gene in both the parent and the final evolved strain to test for possible mutations abolishing its function. However, the sequences were found to be identical in both strains.

Co-fermentation in rich YP medium containing 36 g/L glucose and 37 g/L D-xylose was used to compare the performance of the evolved strain, GS1.11-26, with that of the parent strain HDY.GUF5 (Figure 5b,c). In this condition, both glucose and D-xylose were almost completely consumed in about 13 h by the evolved strain, resulting in a high overall ethanol productivity of 1.4 g/g DW/h (Figure 5b). Compared to the parental strain, the evolved strain showed an 8.5-fold faster rate of D-xylose consumption: 1.10 versus 0.13 g/g DW/h (Table 1). However, the maximum specific glucose consumption rate was slightly higher in the parent strain (1.4-fold) (Table 1). Although the overall ethanol yield per consumed sugar was the same in both strains, the ethanol yield per initial sugar present in the medium was about 2-fold higher for the evolved strain compared to the parent strain (Table 1). This is due to the fact that after 32 h only 5% of the D-xylose was consumed by the parent strain. In the evolved strain, D-xylose consumption started from the beginning of the fermentation (Figure 5b) but remained slow as long as there was glucose present (the first 5 h). The co-consumption of D-xylose during glucose fermentation occurred at a rate of 0.4 g/g DW/h. Afterwards, D-xylose consumption strongly accelerated and attained a volumetric rate only slightly lower than that of glucose consumption. However, the biomass also increased significantly during the glucose fermentation period, so that more biomass was present during the period of fastest D-xylose consumption compared to the period of fastest glucose consumption. As a result, the maximum specific glucose consumption rate (2.71 ± 0.04 g/g DW/h) was about 2.5 times faster than the maximum specific D-xylose consumption rate (1.10 ± 0.00 g/g DW/h), which was reached just after glucose was completely exhausted (Table 1).

**Table 1:** Comparison of fermentation performance between the parent strain, HDY.GUF5, and the evolved strain, GS1.11-26, in YP medium containing a glucose and D-xylose mixture

**Strain**	**Maximum specific sugar consumption rate**		**Yield**			**Specific ethanol productivity**
			**(g/g sugar)**^**a**^			

	**(g/g DW/h)**					**(g/g DW/h)**
	**Glucose**	**D-Xylose**	**Ethanol**	**Xylitol**	**Glycerol**	
HDY.GUF5	3.83 ± 0.08	0.13 ± 0.01	0.23 ± 0.00	0.03 ± 0.00	0.04 ± 0.00	1.79 ± 0.08
GS1.11-26	2.71 ± 0.04	1.10 ± 0.00	0.46 ± 0.00	0.04 ± 0.00	0.06 ± 0.00	1.38 ± 0.01

Note: ^a^: The yield of ethanol and glycerol was calculated per g total sugar while yield of xylitol was calculated per g D-xylose.

### Fermentation performance of strain GS1.11-26 in lignocellulose hydrolysates

#### Separate hydrolysis and fermentation (SHF)

**Figure 6:**
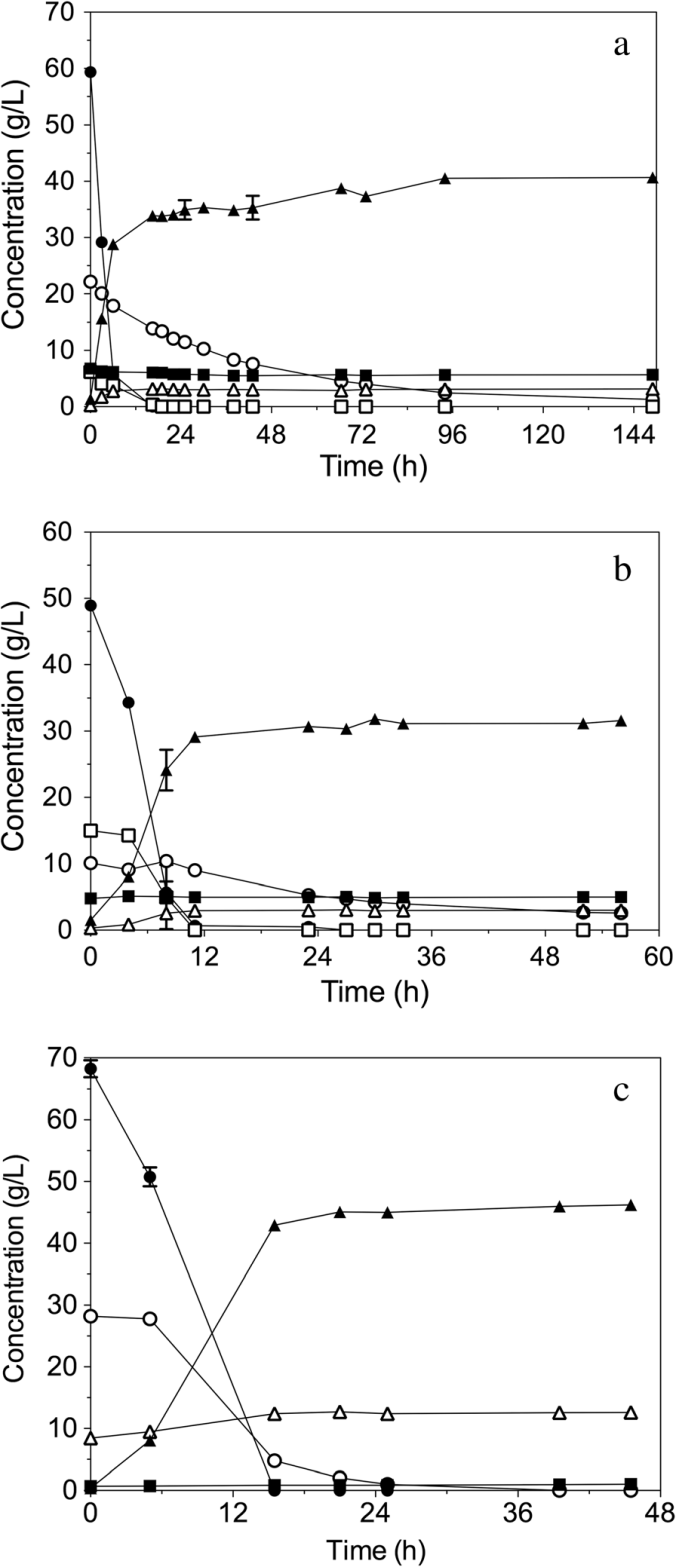
**Performance of strain GS1.11-26 in semi-anaerobic batch fermentations with three different lignocellulose hydrolysates.** (**a**) *Arundo donax* (giant reed), (**b**) spruce and (**c**) mixture of wheat straw/hay. (●) Glucose, (○) D-xylose, (▲) ethanol, (Δ) glycerol, (■) acetate, (□) Mannose.

The fermentation performance of the strain GS1.11-26 was evaluated in hydrolysates of three industrially relevant feedstocks: *Arundo donax* (giant reed), *Picea abies* (Norway spruce), and a 50/50 mixture of wheat straw and hay (Figure 6). Pretreated materials of giant reed and spruce were hydrolyzed using the enzyme cocktail ACCELLERASE® 1500 at 53°C for 48 h. Pretreated material of the wheat straw/hay mixture was hydrolyzed with Novozymes cellulase complex and β-glucosidase (Novozymes A/S; Bagsvaerd, Denmark) at 50°C for 24 h. The whole slurry obtained after hydrolysis was used to start the fermentation. Yeast extract (10 g/L) and peptone (20 g/L) were added as a source of nitrogen, vitamins, amino acids and other nutrients.

In all three hydrolysates tested, a high yield of ethanol, close to the theoretical maximum was obtained with the GS1.11-26 strain (Table 2). The highest yield of ethanol was obtained from the wheat straw/hay hydrolysate, reaching 0.48 g/g of glucose and D-xylose, which is equivalent to 94% of the maximum theoretical yield. In this hydrolysate, no xylitol and only a small amount of glycerol and acetate were detectable at the end of the fermentation, which might have contributed to the high yield.

**Table 2:** Composition of the three lignocellulose hydrolysates and fermentation yield of the evolved strain, GS1.11-26

**Medium**	**Initial sugar level**			**Initial inhibitor concentration (g/L)**			**Yield**		
	**(g/L)**						**(g/g sugars)**		
	**Glucose**	**D-xylose**	**Mannose**	**Acetate**	**HMF**	**Furfural**	**Ethanol**	**Xylitol**	**Glycerol**
*Arundo donax*	59.34	22.18	6.19	6.80	0.32	0.14	0.47 ± 0.01	0.07 ± 0.00	0.04 ± 0.00
Spruce	48.91	10.09	14.97	4.79	1.09	1.57	0.43 ± 0.00	0.003 ± 0.000	0.04 ± 0.00
Wheat straw/hay	68.22	28.22	ND	0.70	ND	ND	0.48 ± 0.02	ND	0.04 ± 0.00

*ND* Not detectable.

*Arundo donax* hydrolysate contained the highest acetate concentration from the three hydrolysates tested. An acetate concentration of about 4 g/L at low pH is known to be inhibitory to growth and fermentation of yeast [39,40]. Moreover, D-xylose fermentation is more sensitive to acetate [41,42]. Despite the presence of an initial acetate concentration of 6.8 g/L in the *Arundo donax* hydrolysate used, the strain consumed all the glucose and more than 90% of the D-xylose in about 96 h with an ethanol yield of 0.47 g/g total sugar, equivalent to 92% of the maximum theoretical ethanol yield (Figure 6a) (Table 2). A final ethanol titer of 4.1% (v/v) was reached in 96 h.

The D-xylose consumption rate was slower in spruce hydrolysate, possibly due to the high concentration of 5-hydroxymethyl-2-furaldehyde (HMF) and furfural in addition to the high acetate level. The initial level of D-xylose was lower than in the other two hydrolysates (Figure 6b), (Table 2). Acid pretreated spruce has been reported to be among the most-inhibitory hydrolysates. It contains high concentrations of phenolic compounds, weak acids and furan derivatives, that synergistically inhibit yeast growth and fermentation [8,43]. Although we measured only the furans and acetic acid, the concentration of these compounds, especially HMF and furfural, was much higher in spruce than in the other two hydrolysates. Furfural and HMF were completely consumed in 4 h, while acetate remained in the medium. In spite of the elevated inhibitor concentrations, a high ethanol yield of 0.43 g/g initial soluble sugars was produced. This is remarkable in view of the high inhibitor concentration.

The wheat straw/hay hydrolysate contained the highest initial sugar concentration and the lowest level of inhibitors (Table 2). Both glucose and D-xylose were completely consumed in about 24 h producing a final ethanol concentration of 45.07 ± 0.92 g/L (Figure 6c), equivalent to 5.8% (v/v). This was the highest ethanol concentration of all three hydrolysates. The rapid fermentation and complete sugar attenuation in wheat straw/hay hydrolysate is likely due to the low level of inhibitors (Table 2).

**Figure 7:**
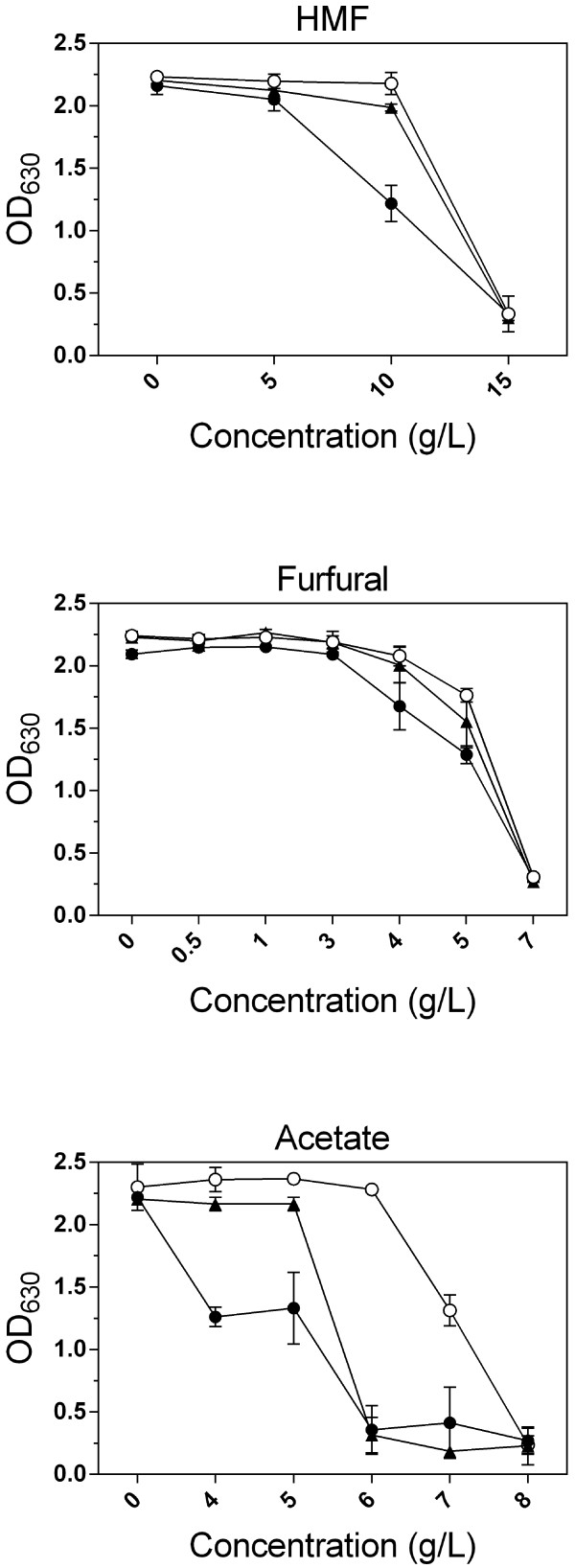
**Evaluation of strain GS1.11-26 for tolerance to HMF, furfural and acetic acid.** Growth assay was performed in 24-well plates containing 1 ml of synthetic complete medium with various concentrations of each inhibitor. Strains were inoculated at an initial OD_630_ of 0.2. Error bars represent standard deviation from duplicate experiments. Strains: (●) GS1.11-26, (○)parent HDY.GUF5, (▲) M315.

Next, we evaluated the tolerance of the GS1.11-26 strain, in comparison with its parent strain HDY.GUF5, to individual inhibitors: HMF, furfural and acetic acid. The strains were inoculated in synthetic medium with glucose and pH 4.5, in the presence of a range of concentrations of the inhibitors. In this condition, the evolved strain GS1.11-26 showed similar tolerance to HMF and furfural, showing no inhibition up to 10 g/L HMF and 5 g/L furfural. However, the tolerance of the evolved strain to acetic acid was reduced (growth up to 5 g/L acetate as measured after 72 h) compared to the parent strain (growth up to 7 g/L acetate as measured after 72 h) (Figure 7).

#### Simultaneous saccharification and fermentation (SSF)

**Figure 8:**
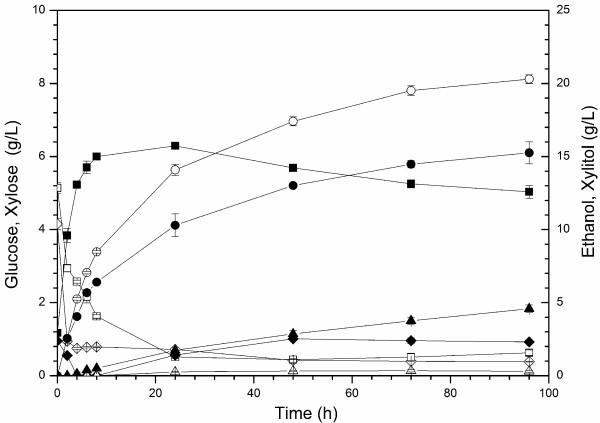
**Performance of strain GS1.11-26 in SSF with pretreated Arundo.** Medium composition during SSF of pretreated Arundo (10% WIS) using GS1.11-26 (open markers). As a comparison, previously reported SSF values [45] with the strain Ethanol Red (filled markers) are shown. Symbols used: glucose (diamond), ethanol (circle), xylose (square) and xylitol (triangle). A yeast concentration of 4 g DW/L was used and a temperature of 39°C. The enzymes used were Celluclast and Novozyme 188.

The performance of the evolved strain was also tested in simultaneous saccharification and fermentation using both pretreated Arundo and spruce material. In a previous study, the yields obtained for SHF and SSF with pretreated Arundo were compared using the XR/XDH strain VTT C-10880 [44]. In that study, the overall yield in SHF was higher than that obtained in SSF. The main reason was most likely that SSF was performed at a temperature of 32°C, which resulted in a low degree of enzymatic hydrolysis. This was confirmed in a study in which SSF was run at both 32 and 39°C with the strain Ethanol Red [45]. For this reason it was decided to assess the strain GS1.11-26 in SSF at 39°C with Arundo hydrolysate and to compare with the results for Ethanol Red reported in the previous study [45]. Due to the favorable D-xylose to glucose ratio [46], D-xylose was consumed already from the beginning of the SSF, remained below 1 g/L from 24 h to 96 h and the xylitol formation was negligible (Figure 8).

The strain GS1.11-26 was also tested in SSF using pretreated spruce. Although the xylan content of spruce is much lower than that of Arundo, the spruce material provided a suitable test of inhibitor tolerance, and SSF experiments were made at both 32 and 39°C. D-xylose consumption was more efficient at 32°C with less than 1 g/L of D-xylose and xylitol at the end of 96 h in the case of spruce (cf. Table 3). The fermentation rate was reduced after 48 h at 39°C (data not shown), and higher amounts of residual glucose and D-xylose were found after 96 h (Table 3). The optimum temperature for SSF was thus lower for spruce than for Arundo.

**Table 3:** Final sugar and metabolite concentrations measured after 96 h SSF with the strain GS1.11-26 using steam pretreated spruce

	**SSF temperature**	
	**32°C**	**39°C**
Residual glucose (g/L)	0.79	3.69
Residual xylose (g/L)	0.40	2.26
Xylitol (g/L)	0.70	0.35
Final ethanol concentration	35.3	30.6
Yield (g ethanol/g total sugar)^*^	0.32	0.28
% of maximum yield	63.9	55.7

A WIS content of 10% was used in the experiments.

*Includes only fermentable sugars from fiber and liquid fractions.

### Performance of strain GS1.11-26 in high-density fermentation

**Figure 9:**
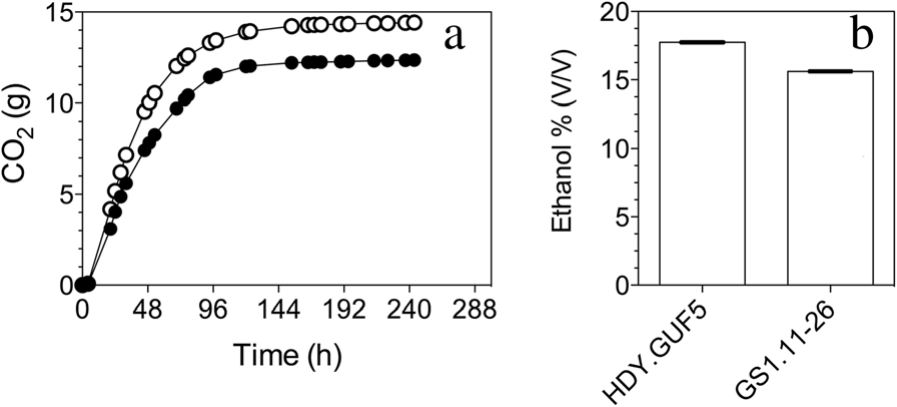
**Performance of strain GS1.11-26 in high gravity fermentation.** (**a**) CO_2_ production as measured by weight loss in semi-anaerobic batch fermentations in YP + 330 g/L glucose at 30°C. (●) strain GS1.11-26, (○) parent strain HDY.GUF5. (**b**) Final ethanol titer reached in the fermentations of (**a**). Each experiment was performed in duplicate, and error bars represent standard deviation from the average of duplicate values.

We have tested strain GS1.11-26 for tolerance to high osmolarity and high ethanol in very high gravity (VHG) fermentations. This is a challenging quality test for industrial yeast, because it requires a combination of very high osmotolerance and very high ethanol tolerance [47,48]. The original, untransformed parent strain Ethanol Red shows very good performance in unstirred, semi-anaerobic VHG fermentations, accumulating about 17-18% ethanol from 330 g/L glucose in YP medium (not shown) [48]. The same performance was observed in the genetically modified parent strain HDY.GUF5 (Figure 9a and b). On the other hand, the evolved strain, GS1.11-26, showed a slower fermentation rate and a 1-2% (v/v) lower final ethanol titer under the same VHG conditions (Figure 9a and b). This indicates that adverse background mutations have been introduced in the evolved strain during the EMS treatment or spontaneously generated during the evolutionary engineering.

**Figure 10:**
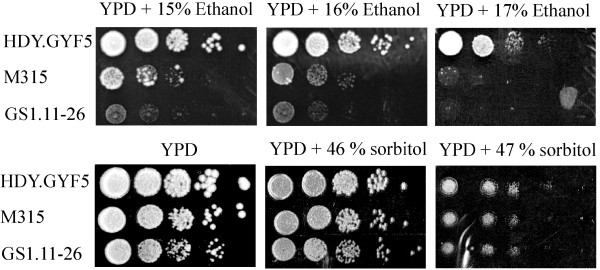
**Effect of ethanol and osmotic stress on growth of GS1.11-26.** Growth on YPD plates containing different concentrations of ethanol or sorbitol was performed with a spot assay in 10-fold serial dilutions from an initial OD_600_ value of 0.5. The assay was performed two times with similar results using independent cultures.

To evaluate whether the slow fermentation performance in VHG fermentation is due to impaired tolerance to osmotic or ethanol stress, we examined the tolerance of the evolved strain to both stresses in comparison to the parent strain. Figure 10 shows the results of a growth test by dilution spot assays on solid YPD medium containing a high concentration of sorbitol (for osmotic stress) or a high concentration of ethanol. Though the tolerance to osmotic stress by the evolved strain GS1.11-26 was similar to that of the parent strain HDY.GUF5, the ethanol tolerance was severely reduced in the evolved strain, which was manifested by moderate growth at an ethanol concentration of only 14% (v/v). The parent strain was able to grow at an ethanol concentration of up to 17% (v/v).

### Growth rate of strain GS1.11-26 under aerobic conditions

The production of a maximal amount of yeast cell biomass under aerobic conditions is one of the requirements for industrial yeast propagation. The latter is performed in highly-controlled aerobic fed-batch fermentation in which the sugar level is maintained at a very low level (below 0.1% w/v) to avoid any production of ethanol. Hence, the yeast should be able to combine a high growth rate with a purely respiratory metabolism to maximize the production of biomass [49]. With this notion, the strain GS1.11-26 was evaluated for growth rate under aerobic conditions in different media and volumes. In a bioscreen assay with synthetic medium containing 20 g/L glucose, the maximum respiro-fermentative growth rate of the evolved strain, GS1.11-26, was only about 75% (0.342 ± 0.005 h^-1^) of that of the parent strain, HDY.GUF5, (0.459 ± 0.021 h^-1^). Moreover, the mutants M315 and M492, which were used for the genome shuffling step, grew faster than GS1.11-26 and slower than HDY.GUF5. This might indicate that the genetic changes causing the slower aerobic growth rate in GS1.11-26 occurred both during mutagenesis and the subsequent genome shuffling and/or evolutionary adaptation process.

**Figure 11:**
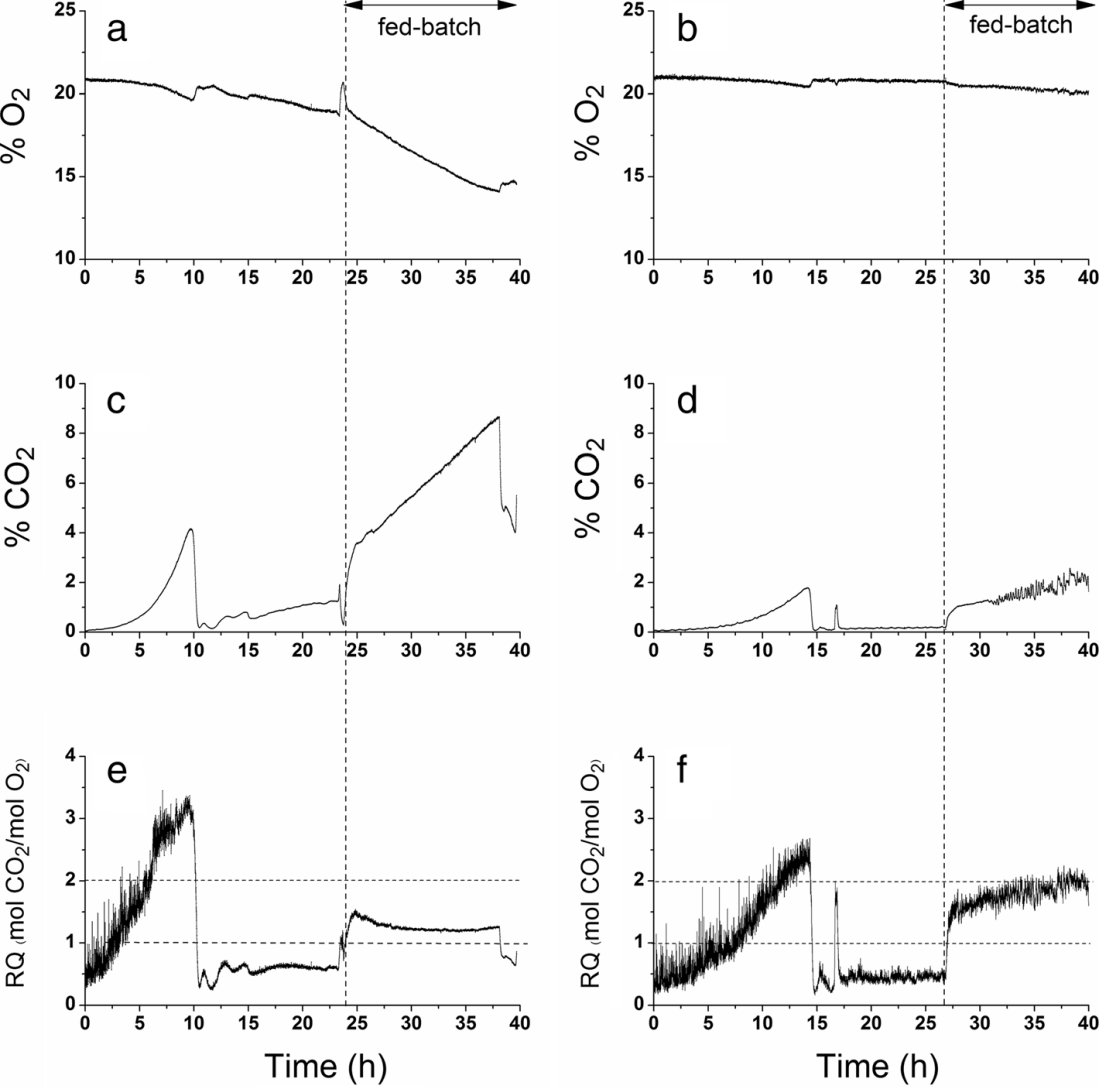
**Evaluation of GS1.11-26 performance under aerobic conditions.** Comparison of exit gas profiles of Ethanol Red (left panels) and GS1.11-26 (right panels) during aerobic batch (synthetic medium) and fed-batch cultivation (arundo hydrolysate). (**a,b**) percent O_2_ in exit gas, (**c,d**) percent CO_2_ in exit gas, and (**e,f**) measured respiratory quotient (mol CO_2_/mol O_2_).

**Figure 12:**
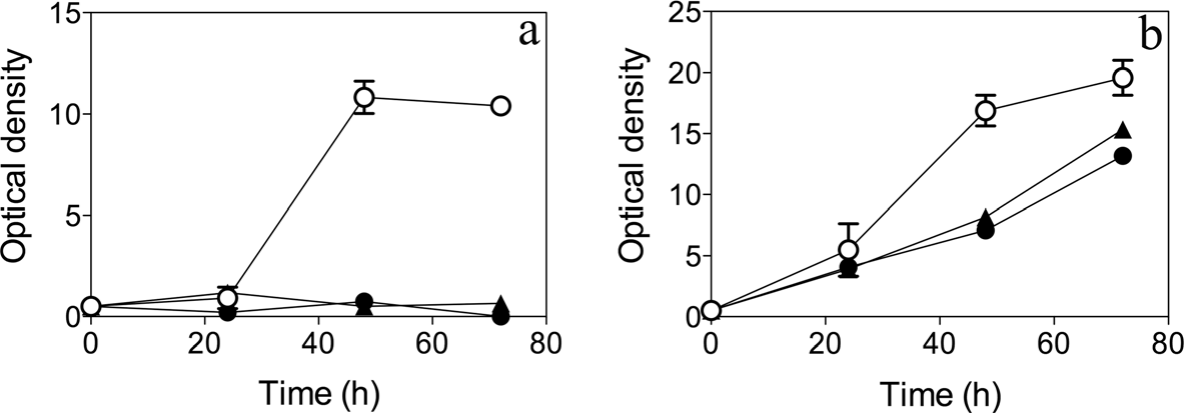
**Growth rate of strain GS1.11-26 under aerobic conditions.** Growth in shake flask cultures at 30°C with YP medium containing 5% (v/v) ethanol (**a**) or 5% (v/v) glycerol (**b**) as sole carbon source. Strains were inoculated at an initial OD_600_ of 0.5 and tested in duplicate. The OD_600_ was normalized by subtracting growth in YP medium without added carbon source. Error bars represent standard deviation from duplicate experiments. Strains: (●) GS1.11-26, (○) parent HDY.GUF5, (▲) M315.

In shake flask cultures with rich medium (40 ml YPD), a similar difference between the evolved and parent strain was observed. The aerobic respiro-fermentative growth rate of the evolved strain, GS1.11-26, was only 80% (0.485 ± 0.032 h^-1^) of that of the parent strain, HDY.GUF5, (0.614 ± 0.096 h^-1^). Moreover, there was no second growth phase after the diauxic shift in the evolved strain, which was confirmed in aerobic batch cultivation (Figure 11). Hence, the evolved strain was apparently unable to utilize the ethanol produced after the glucose in the medium was exhausted. The absence of growth on ethanol in the evolved strain was further confirmed with a growth assay in liquid YP medium containing 5% ethanol as a sole carbon source (Figure 12a). Furthermore, it was not possible to obtain respiratory growth on glucose with GS1.11-26 in a fed-batch cultivation on Arundo hydrolysate, in contrast to what was found for the parental strain Ethanol Red (Figure 11). On the other hand, the evolved strain was able to grow in medium containing glycerol as sole carbon source, but with a slower growth rate than the parent strain (Figure 12b). This indicates that the strain was not completely defective for respiration, but that its maximal rate of respiration was significantly reduced compared to the parent strain. The complete lack of growth in ethanol might be due to a specific additional inability of the strain to metabolize ethanol as a carbon source.

### Stability of the D-xylose fermentation phenotype in strain GS1.11-26

**Figure 13:**
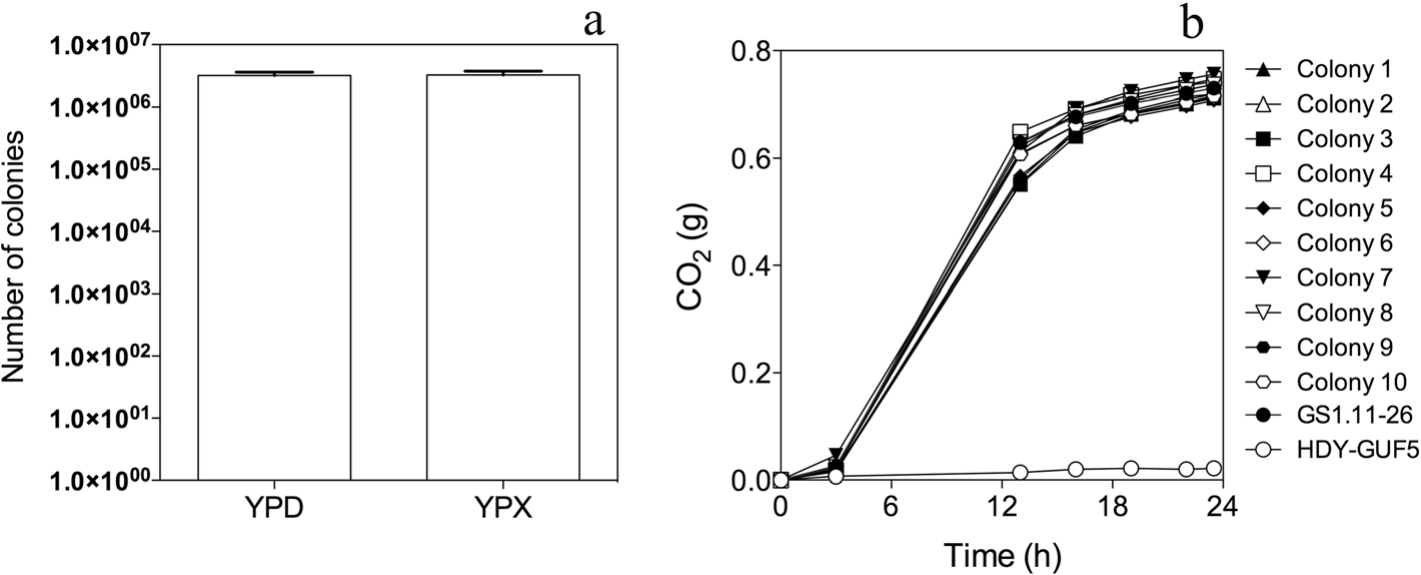
**Evaluation of the stability of D-xylose fermentation capacity in strain GS1.11-26.** (**a**) Comparison of the number of colonies growing in YPD and YPX after growth for about 50 generations in non-selective conditions with only glucose as carbon source. Error bars represent standard deviation from three independent experiments. (**b**) Fermentation performance in YP + 40 g/L D-xylose as estimated from weight loss due to CO_2_ release. Colonies 1 to 10 represent randomly selected single-cell clones isolated after growth of GS1.11-26 for 50 generations in YPD medium. The parent strain HDY.GUF5 is shown for comparison.

We have assessed the stability of the D-xylose fermentation phenotype through many generations of growth in rich glucose medium in the absence of any D-xylose. For that purpose, three independent colonies of strain GS1.11-26 were inoculated into 5 ml YPD and serially transferred, at 100 times dilution each, for about 50 generations. Subsequently, replicate samples were spread onto YPD and YPX plates and the ratio of the number of colonies growing on YPX relative to that on YPD was calculated. As shown in Figure 13a, the number of colonies on the YPD and YPX plates was very similar. Moreover, there was no apparent difference in the size of the colonies growing on the YPX plates. When ten randomly selected single-cell clones, obtained from the last YPD culture, were tested for fermentation performance in YPX, all ten showed a fermentation performance very similar to that of the original GS1.11-26 strain (Figure 13b). These results indicate that the D-xylose fermentation capacity of the GS1.11-26 strain was completely stable in the absence of any selection pressure.

### Enhanced D-xylose isomerase activity in strain GS1.11-26

**Figure 14:**
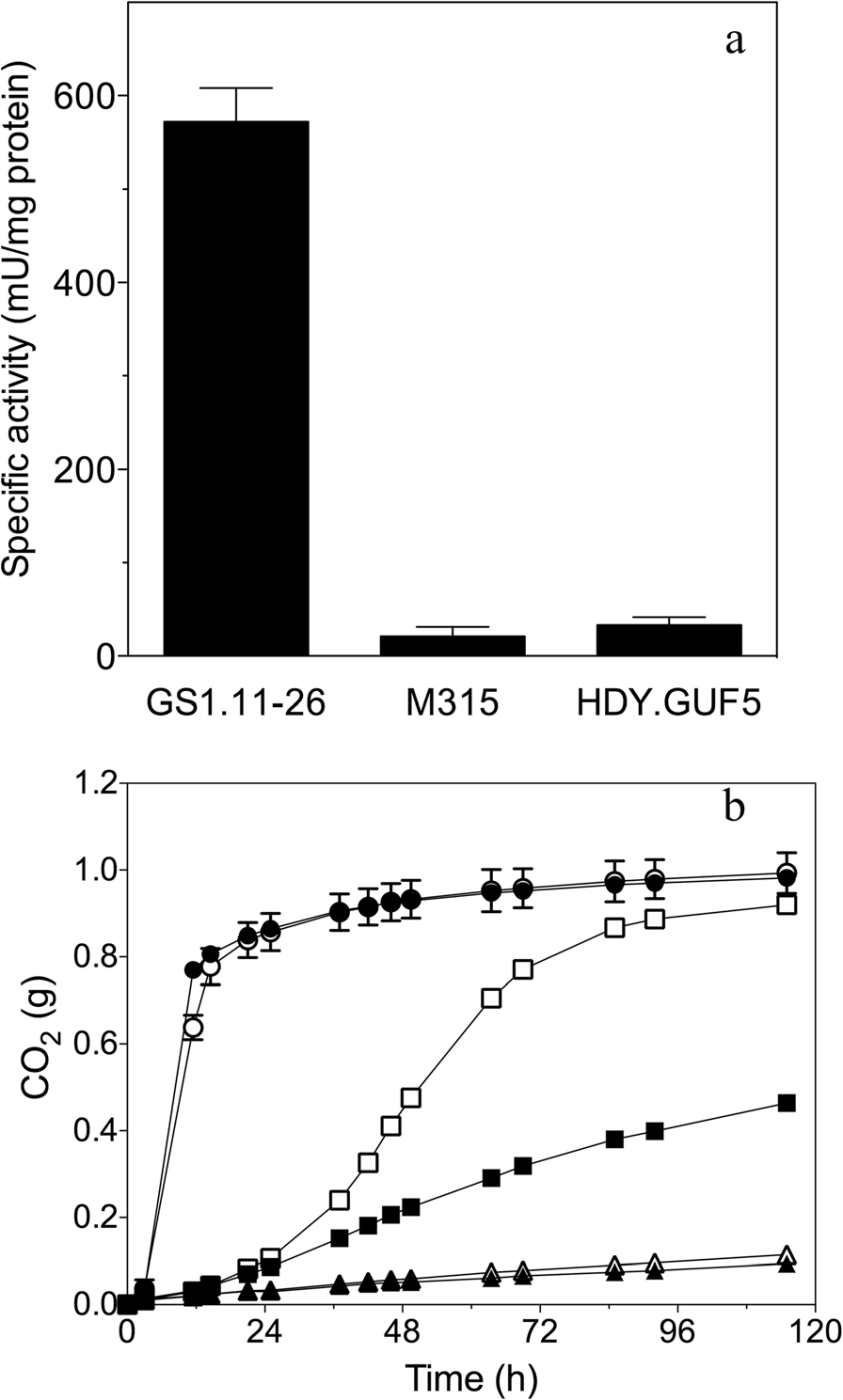
**D-xylose isomerase activity and its effect on D-xylose fermentation capacity.** (**a**) Specific XI activity as measured in cell extracts of strains GS1.11-26, M315 and HDY.GUF5. M315 was obtained after the EMS mutagenesis procedure of parent strain HDY.GUF5. Error bars represent the standard deviation from triplicate experiments. (**b**) Effect of *XylA*, encoding XI, overexpression from a multi-copy plasmid on D-xylose fermentation capacity. Fermentation performance in YP + 40 g/L D-xylose was estimated from weight loss due to CO_2_ release. The experiment was repeated with at least three independent transformants. Error bars represent standard deviations from the average of two independent transformants. Strains: (●) GS1.11-26, (○) GS1.11-26 + pXI, (▲) HDY.GUF5, (∆) HDY.GUF5 + pXI, (■) M315, (□) M315 + pXI.

Previous work has shown that evolutionary engineering for improved growth on D-xylose in a laboratory strain led to increased activity of the heterologous *Piromyces* XI expressed from a plasmid, although the precise molecular cause for the increase was not identified [50]. To test whether a similar change might have occurred in our strain, we measured XI activity in cell extracts of the final evolved strain GS1.11-26. We found a dramatic, 17-fold increase in specific XI activity compared to the parent strain HDY.GUF5 (Figure 14a). Sequence analysis of the heterologous gene *XylA,* coding for XI, in the evolved strain GS1.11-26 and in the parent strain HDY.GUF5, did not reveal any nucleotide polymorphism. Hence, the increase in the XI activity does not seem to be due to a change in the intrinsic activity of the enzyme. It might be due to amplification of the gene in the evolved strain.

The very strong increase in XI activity might be a major reason for the improved D-xylose fermentation rate in the GS1.11-26 strain. The final XI specific activity of 0.57 U/mg protein is in the same range as previously reported values for evolutionary engineered strains expressing fungal *Piromyces* XI [50,51]. The M315 mutant, selected after the mutagenesis procedure, did not show any increase in XI activity compared to the parent strain HDY.GUF5 (Figure 14a).

To assess the importance of the enhanced XI activity, we overexpressed the original *Clostridium phytofermentans XylA* gene construct on a multi-copy plasmid in the parent strain, HDY.GUF5, and the M315 mutant selected after the mutagenesis procedure. There was no improvement in the D-xylose fermentation rate in transformants of HDY.GUF5 (Figure 14b). On the other hand, the M315 mutant with the *XylA* overexpression plasmid showed a strong improvement in the D-xylose fermentation rate, although the rate was still much lower than that of the final evolved strain, GS1.11-26 (Figure 14b). This indicates that high XI activity is indeed beneficial for D-xylose fermentation but that it requires one or more mutations in the genome of the strain, which were introduced during the mutagenesis procedure, to be effective. In strain GS1.11-26 additional overexpression of *XylA* did not result in further improvement in the D-xylose fermentation rate, indicating that XI activity is no longer limiting the fermentation in this evolved strain under the experimental conditions used.

## Discussion

The XI from *C. phytofermentans* was the first prokaryotic XI that showed high activity upon expression in *Saccharomyces cerevisiae,* both in laboratory and industrial strains [27]. However, the industrial strain expressing the codon-optimized version of the gene could only ferment D-xylose to ethanol after further evolutionary adaptation in D-xylose medium. Though the rate of D-xylose utilization by the evolved strain was much too low to allow industrially viable ethanol production from lignocellulosic feedstocks, the work provided a starting point for the development of strains using a bacterial XI that was less inhibited by xylitol than the fungal *Piromyces* XI. In the present work we combined rational metabolic engineering based on expression of the *C. phytofermentans* XI with systematic evolutionary engineering, and developed a robust industrial *S. cerevisiae* strain that efficiently converts D-xylose to ethanol with high yield and productivity.

Rational metabolic engineering alone was not able to establish efficient D-xylose (or L-arabinose) utilization capacity in the Ethanol Red strain. The recombinant strain HDY.GUF5 failed to show significant D-xylose (or L-arabinose) fermentation. Efficient rational engineering strategies rely on the complete understanding of the metabolic network, as well as its regulation in response to the dynamic environmental conditions to which the engineered strain is exposed [10]. Because of the complexity and still limited understanding of the biological and regulatory network of D-xylose metabolism in recombinant *S. cerevisiae* strains, rational approaches have faced huge challenges to eliminate the factors that limit efficient D-xylose fermentation [52]. Several of these factors have been identified [31,52]. Most of these requirements have been addressed in the strain HDY.GUF5, which include overexpression of the PPP genes, *XKS1* and the hexose/pentose transporter encoding gene *HXT7*, as well as codon optimization of some of the genes based on the highly efficient glycolytic codon usage of yeast [33]. Although expression of the same codon-optimized XI in a laboratory strain established moderate D-xylose fermentation [27], the industrial strain used in this study as well as previously [27], was not able to metabolize D-xylose. This is likely due to the difference in the genetic background of the strains, although the precise mechanism remains unclear [53].

Combining metabolic engineering with evolutionary engineering alone or together with random mutagenesis has been proven successful for developing strains with improved D-xylose fermentation efficiency [20,29,51,54,55]. In addition, genome shuffling has also been used in combination with metabolic engineering and evolutionary adaptation, for improving D-xylose utilization capacity in different *S. cerevisiae* strains [56,57]. In the present paper, we successfully exploited a combinatorial approach using all three random strain improvement strategies described above, in order to improve D-xylose fermentation efficiency of the recombinant industrial strain HDY.GUF5.

We first started with random mutagenesis of the recombinant strain to generate very diverse genetic variation that might establish initial D-xylose fermentation capacity. Selection of mutants with a significant D-xylose anaerobic fermentation rate is a challenging task, because likely multiple mutations are required [10]. In addition, a previous study reported that direct selection of a mutant *S. cerevisiae* population capable of anaerobic D-xylose utilization, was unsuccessful [55]. Therefore, we first selected clones from a heavily-mutagenized population that were able to grow at least to some extent on D-xylose medium as a sole carbon source. Since strong random mutagenesis likely results in both beneficial and deleterious mutations, we presumed that genetic recombination of the mutants obtained, with the original industrial strain by genome shuffling, and selection for D-xylose utilization capacity, would result in enrichment of beneficial and loss of unfavorable mutations [58]. After only one step of genome shuffling, the whole shuffled culture already demonstrated a significantly improved rate of D-xylose fermentation. However, attempts to isolate single cell clones from this shuffled culture with better D-xylose utilization rate, compared to that of the best mutant strain M315, failed. Thus, we decided to enrich the clones with most rapid D-xylose utilization and at the same time further improve their rate of D-xylose utilization, through adaptive evolution in D-xylose medium. The selection of clones with a shorter lag phase on D-xylose and a higher D-xylose utilization rate is most obvious. However, clones that utilize a larger part of the D-xylose will be able to undergo more proliferation cycles and therefore will tend to be present in higher amounts and thus also preferentially transferred to the next culture. Since the cultures were semi-anaerobic, the D-xylose is largely converted to ethanol and therefore these clones will likely also have a higher ethanol yield.

After only two transfers in D-xylose medium under semi-anaerobic conditions, the D-xylose fermentation rate already increased dramatically. Subsequent serial transfers resulted in further gradual improvement of D-xylose utilization. In previous studies, evolutionary adaptation under aerobic conditions followed by gradual transition to anaerobic conditions, was necessary to obtain strains with anaerobic D-xylose utilization capacity. In addition, several generations were required to obtain strains with efficient D-xylose fermentation capacity [28,55,59]. In our study, even though the best isolate GS1.11-26 was isolated after 11 serial transfers, clones with high D-xylose utilization capacity could already be isolated after only 2 transfers. The rapid improvement in the rate of D-xylose fermentation might be explained by the presence of a suitable combination of important genetic changes introduced by the mutagenesis and genome shuffling, and sustaining rapid improvement of D-xylose utilization by a repetitive subsequent genetic modification, such as amplification of the *XylA* gene or another crucial genetic element, and/or rapid enrichment of clones with a superior combination of mutant alleles. An important genetic change might have been generated also in the second culture of the evolutionary adaptation, which was characterized by a sharp rise in CO_2_ evolution at the end, and a dramatic increase in the rate of fermentation when this culture was transferred to the next batch. During evolutionary engineering, expansions and contractions of different subpopulations can occur [60,61] and an individual cell with a beneficial mutation, providing a relative fitness advantage, can develop into a dominant subpopulation after several generations in serial batch transfer experiments [62]. In our work, high variability in the rate of D-xylose fermentation was observed among individual clones isolated from intermediate cultures in the evolutionary adaptation process. However, isolates from the last culture showed a very similar fermentation performance, although not precisely the same, suggesting that the fitter clones finally conquered and dominated the culture.

The best strain, GS1.11-26, showed a reproducible and stable D-xylose fermentation rate and was further characterized both in laboratory medium and in three industrially relevant lignocellulosic feedstocks. In synthetic medium with D-xylose as a sole carbon source, the GS1.11-26 strain showed a maximum specific D-xylose consumption rate at least 15 times higher than the previous industrial strain BWY10Xyl expressing the same codon-optimized *C. phytofermentans* XI [27]. The GS1.11-26 strain also accomplished complete attenuation of D-xylose with an ethanol yield of 0.46 g/g D-xylose, whereas the previous strain BWY10Xyl left a substantial amount of D-xylose unfermented. Moreover, the yield of ethanol obtained with GS1.11-26 was higher than the yield obtained with the best strain reported recently [51]. As a consequence, GS1.11-26 exhibited the highest D-xylose to ethanol conversion yield than any other recombinant strain of *S. cerevisiae* reported so far. The high ethanol yield can also be explained by the very low xylitol yield, which is remarkable since the *GRE3* gene had not been deleted nor was it inactivated in the strain development programme. The low xylitol yield, in the absence of *GRE3* inactivation, might be due to the inherently higher metabolic flux in the industrial bioethanol production strain Ethanol Red, compared to the previously used strain backgrounds.

The GS1.11-26 strain also performed very well in lignocellulose hydrolysates both in SHF and SSF. The yield of ethanol per g consumed sugars, was slightly higher in all the lignocellulose hydrolysates compared to that in synthetic and YP medium. This is probably due to the lower amount of xylitol and glycerol formed, and is consistent with previous results [46,63]. In SHF, it reached high maximum D-xylose consumption rates of 1.1 g/g inoculum DW/h and it showed partial co-fermentation of glucose and D-xylose during separate hydrolysis and fermentation. We have used a parameter for calculation of the specific sugar consumption rate based on the initial inoculum density, since the whole slurry was used for the fermentation experiment and since it is difficult to estimate the biomass during the fermentation process.

The ethanol yield from glucose and D-xylose in lignocellulose hydrolysates was also close to maximum and final ethanol titers between 3.9 and 5.8% (v/v) were reached, depending on the type of hydrolysate. The GS1.11-26 strain maintained a high level of tolerance like its parent Ethanol Red in inhibitor rich spruce hydrolysate and to individual inhibitors HMF and furfural. However, the strain did not retain the same high ethanol tolerance as the original Ethanol Red parent strain, though it was still able to accumulate more than 15% ethanol in very high-gravity fermentation (YP + 330 g/L glucose). The relatively high tolerance of GS1.11-26 to inhibitors, like HMF and furfural, found in spruce hydrolysate, but its lower tolerance to other stresses, like ethanol and acetic acid, can be explained by the fact that, after the genome shuffling step spruce hydrolysate was used as selective medium. The cells that were able to grow in spruce hydrolysate were further used for the evolutionary adaptation. This result demonstrates the importance of the selection conditions during evolutionary engineering, which is in agreement with the principle, “you get what you screened for” [64]. GS1.11-26 showed reduced tolerance to acetic acid compared to the parent HDY.GUF5. This can be explained by different mechanisms underlying tolerance to various inhibitors [61]. In *S. cerevisiae*, the tolerance mechanism to HMF and furfural, is similar, but distinct from that of acetic acid [65].

SSF is an interesting process for production of ethanol from lignocellulosic feedstocks, e.g. because it strongly reduces feedback-inhibition on enzymatic hydrolysis by the liberated monosaccharides and also reduces the danger of contamination. SSF performed at higher temperature (39°C) was also shown to increase the final yield of ethanol, because of a better compromise between the temperature optima of the enzymes and the yeast [45]. In this respect, GS1.11-26 performed very well with almost complete attenuation of both glucose and D-xylose in about 96 h at 39°C. In a previously reported SSF of Arundo hydrolysate using Ethanol Red [45], there was also some D-xylose consumption, but only as a result of D-xylose reduction to xylitol. In our study, the final ethanol concentration for GS1.11-26 was 20.3 g/L, corresponding to an ethanol yield of 0.29, to be compared to the previously reported values for Ethanol Red of 15.3 g/L and 0.22 g ethanol/g total sugar. The ethanol yield thus increased by about 32% due to the efficient D-xylose conversion. The increase in the ethanol yield was in fact slightly higher than the increase expected from the D-xylose conversion alone, possibly due to removal of D-xylose inhibition on enzymatic hydrolysis.

Cell extracts of strain GS1.11-26 displayed 17-fold higher XI activity compared to cell extracts of the parent strain. However, there were no mutations in the *XylA* gene. Increased XI activity without any mutations in the *XylA* gene has also been reported recently [50,51]. The high XI activity was explained by integration into the genome in multiple copies of the plasmid carrying the *XylA* gene [51]. Although the recombinant strain in our work has been constructed through chromosomal integration of the *XylA* gene, it is possible that multiple chromosomal amplifications of the gene have occurred during the evolutionary adaptation process. The precise mechanism behind the establishment of the high XI activity in GS1.11-26 is currently being investigated and will be reported elsewhere.

Overexpression of XI in the parent strain did not increase the D-xylose consumption rate, as opposed to overexpression in the mutant strain M315 obtained after the mutagenesis step. This indicates that rapid D-xylose consumption requires a synergistic interaction between high XI activity and one or more mutations in the genome, which is in agreement with another report, in which high D-xylose assimilation capacity could only be attributed partially to the high activity of XI [51].

This clearly shows that the generation of other genetic changes, e.g. as obtained in our work by chemical mutagenesis, is essential for development of a pentose-utilizing strain with high performance. On the other hand, the random mutagenesis steps also resulted in unfavorable effects on other properties, such as reduced aerobic growth rate in glucose and a reduced glucose fermentation rate. This has also been reported previously during selection of a recombinant *S. cerevisiae* strain for anaerobic growth in D-xylose. In that report, strains exhibiting significant improvement in anaerobic D-xylose utilization also showed a reduced aerobic growth rate in glucose [55]. We do not know whether the reduced glucose consumption rate or reduced aerobic growth rate in glucose, are trade-offs for the high D-xylose utilization capacity. Future research will have to show whether these negative side-effects are due to background mutations in the strain, which can be lost without affecting its high performance for D-xylose utilization, or whether they are causally linked to the high D-xylose fermentation rate. This will have important implications for further improvement of the strain for efficient co-fermentation of glucose, D-xylose and L-arabinose. In spite of this, the GS1.11-26 shows highly promising potential for further development of an all-round robust yeast strain for efficient fermentation of various lignocellulose hydrolysates. Moreover, it already contains the genes for additional utilization of L-arabinose and should be easily evolved also for efficient fermentation of this pentose sugar.

## Conclusions

We have developed a robust industrial *S. cerevisiae* strain exhibiting the highest yield of ethanol from D-xylose. The high D-xylose fermentation capacity was completely stable after many generations of growth in the absence of D-xylose. High activity of XI was found to be the main but not the only reason for fast D-xylose assimilation capacity. The final evolved strain also demonstrated an efficient fermentation rate of glucose and D-xylose in inhibitor-rich lignocellulose hydrolysates. However, the evolved strain GS1.11-26 showed a partial respiratory defect causing a reduced aerobic growth rate and it also had a slightly reduced glucose fermentation rate. GS1.11-26 has a significant potential for further development of a robust industrial yeast strain for bioethanol production with various lignocellulose hydrolysates.

## Methods

### Strains and growth conditions

The *S. cerevisiae* strains utilized in this study are listed in Table 4. Yeast cells were propagated in yeast extract peptone (YP) medium (10 g/L yeast extract, 20 g/L bacteriological peptone) supplemented with either 20 g/L D-xylose (YPX) or 20 g/L D-glucose (YPD). For solid plates, 15 g/L Bacto agar was added after adjusting the pH to 6.5. For batch fermentation, either complex YP medium or synthetic complete medium (1.7 g/L Difco yeast nitrogen base without amino acid and without ammonium sulfate, 5 g/L ammonium sulfate, 740 mg/L CSM-Trp and 100 mg/L L-tryptophan) supplemented with D-xylose or D-glucose/D-xylose mixture was used. For selection of strains expressing a multi-copy plasmid containing *Kan*MX resistance marker, 200 mg/L geneticin was added to the medium. Yeast strains were maintained at −80°C in stock medium composed of YP and 30% glycerol.

**Table 4:** ***S. cerevisiae *strains used in the study**

**Yeast strain**	**Main characteristics**	**Source/reference**
Ethanol Red	Industrial bioethanol production strain, *MAT****a****/α*	Fermentis, a division of S. I. Lesaffre, Lille, France
HDY.GUF5	Ethanol Red**;***pyk2::XylA*; *XKS1; TAL1; TKL1; RPE1; RKI1; HXT7;AraT; AraA; AraB;AraD; TAL2; TKL2*	This study
M315	HDY.GUF5 + 3 h mutagenesis in 3% EMS, *MATα/α*	This study
M492	HDY.GUF5 + 4 h mutagenesis in 3% EMS, *MAT****a****/α*	This study
GS1.11-26	HDY.GUF5***,*** M315 and M492 + genome shuffling and evolutionary adaptation, *MATα/α*	This study
TMB3400	USM21 HIS3::YIpXR/XDH/XK + mutagenesis and selection	[37]

### Mutagenesis and genome shuffling

Overnight-grown yeast cells were harvested, washed twice with phosphate buffer (pH 7), and re-suspended in 1 ml sodium phosphate buffer pH 7 at a cell concentration of 2 × 10^8^ cells/ml. Five different samples were treated with a final concentration of 3% Ethyl Methanesulfonate (EMS) or only phosphate buffer (as control) for different time intervals at 30°C. The EMS was subsequently neutralized by washing twice with freshly prepared 5% sodium thiosulphate. The cell pellets were then re-suspended in sterile 500 μl milliQ water and plated in aliquots of 100 μl onto both YPX and YPD plates. To estimate the percentage survival after mutagenesis, colonies of EMS treated cells from the YPD plates were counted and the ratio relative to that of untreated cells was calculated.

For genome shuffling, cells selected based on growth on D-xylose and sporulation efficiency were sporulated in 1% potassium acetate medium. After 7 days at 23°C, asci were harvested and spores were purified [66]. The purified spores from each strain were mixed together and allowed to germinate for 2 h in YPD medium. Exponentially growing cells from a ***MAT***α/α diploid strain (M315) were mixed with the germinated spores. The cells were allowed to mate in 40 ml YPD in a shaking incubator at 70 rpm for 48 h. To select D-xylose growing strains, the zygotes were subsequently transferred to YP medium containing D-xylose as a sole carbon source. The D-xylose growing cells were inoculated into the liquid fraction of acid pretreated spruce hydrolysate at three different concentrations (40%, 50% and 60%). To maintain the D-xylose growth phenotype, 40 g/L D-xylose was added to the spruce hydrolysate medium. Cells growing in the highest concentration of hydrolysate were grown again in YP medium containing D-xylose and subsequently used to start the evolutionary adaptation by sequential batch cultivation.

### Determination of mating type

Determination of the mating type was done by PCR and pheromone assay. PCR was performed with a primer for the MAT locus and a *MAT****a ***or *MATα* specific primer [67]. To validate mating type by a pheromone assay, two tester strains of *S. cerevisiae*, *MATa bar1-∆* and *MATα sst2-∆*, were used. A small amount of tester strain was mixed with 1% agar at 50°C and immediately poured on top of a YPD plate. After the top agar solidified, about 10 μl of cell suspension from strains to be tested was spotted onto each tester plate. After 24 h incubation at 30°C, *MATα* cells showed a zone of growth inhibition on plates of the *bar1-∆* strain while ***MATa ***cells showed a zone of growth inhibition on plates of the *sst2-∆* strain. Diploid cells did not produce a zone of inhibition.

### Molecular Biology methods

Yeast cells were transformed with the LiAc/SS-DNA/PEG method [68,69]. Genomic DNA from yeast was extracted with PCI [phenol/chloroform/isoamyl-alcohol (25:24:1)] [70]. Polymerase chain reaction (PCR) was performed with Phusion DNA polymerase (New England Biolabs) for construction of the vectors and sequencing purposes and ExTaq (Takara) or Taq (NEB) for diagnostic purposes. Sanger sequencing was performed by the Genetic Service Facility of the VIB.

### Plasmid construction

Plasmids were propagated in *E. coli* strain TOP10 (Invitrogen) or DH5α (NEB), grown in LB medium, containing 100 μg/ml ampicillin at 30°C or 37°C. *E. coli* cells were transformed using the CaCl_2 _[71] or electroporation method [72]. The plasmids pHD8 and pHD22 were constructed by homologous recombination in yeast from up to 17 single overlapping PCR fragments. The templates used were genomic DNA from *S. cerevisiae*, the plasmids pUG6 [73], pZC1 [74], p426H-i-opt.XI [24], YEparaAsynth and YEparaDsynth [33] as well as the codon-optimized genes of *XKS1* and *E.coli araB* (method described in [33] - Sloning BioTechnology) and NQM1 and TKL2 (DNA2.0). The assembly of the multi-copy plasmid carrying the *XylA* gene, was similar to the plasmid pHD8, but the genes flanked by the i1 and i3 regions (see Figure 1a) were substituted by restriction digestion and ligation with one copy of the *XylA* gene.

### Determination of ploidy by flow cytometry

Flow cytometry analysis of DNA content was performed according to Popolo et al. [75]. Briefly, exponentially growing cells were washed with ice-cold sterile water and fixed with 70% ethanol. Cells were treated with RNase (1 mg/ml) and the DNA was stained with propidium iodide (0.046 M) in 50 mM Tris, pH 7.7 and 15 mM MgCl_2_, at 4°C for about 48 h. The fluorescence intensity was measured using a FACScan instrument (Becton Dickinson).

### Determination of specific D-xylose isomerase activity

The specific activity of D-xylose isomerase was measured based on the isomerization of D-xylose to xylulose, followed by reduction of xylulose to xylitol by sorbitol dehydrogenase [76]. Cell extraction was performed by disruption with glass beads using a Fast Prep homogenizer. Protein concentration was determined using the Pierce 660 nm Protein Assay kit (Thermo Scientific) according to the manufacturer’s manual. XI activity in the fresh cell extract was determined at 30°C. The assay mixture contained 100 mM Tris–HCl buffer (pH 7.5), 10 mM MgCl_2_, 0.15 mM NADH and 2U sorbitol dehydrogenase. The reaction was started by addition of D-xylose to a final concentration of 500 mM. A molar extinction coefficient of 6.25 (mM cm)^-1^ at 340 nm for NADH was used to calculate specific activity. Specific activity was expressed as Units per mg protein. One unit corresponds to the conversion of 1 μM of substrate into product in one min under the specified reaction conditions.

### Small-scale fermentations

Semi-anaerobic sequential batch fermentations were performed in 100 ml YP medium containing 40 to 100 g/L D-xylose as sole carbon source, in cylindrical tubes with cotton plugged rubber stopper and glass tubing. Cultures were continuously stirred magnetically at 120 rpm and incubated at 35°C. Semi-anaerobic batch fermentations in synthetic or complex medium were performed in 300 ml shake flasks with a working volume of 200 ml at 35°C. Flasks were closed with fermentation locks containing glycerol. Nitrogen gas was sparged after cell inoculation until the oxygen concentration reached about 2 ppm. Cultures were continuously stirred at 120 rpm using a magnetic stirrer. Samples were taken every few hours with needles.

### Inhibitor tolerance assay

Tolerance to osmotic and ethanol stress was performed with solid medium [77,78]. Strains were inoculated in YPD medium and grown at 30°C for 2 days until stationary phase. Cultures were diluted to an OD_600_ of 0.5, and 5 μl of a twofold dilution was spotted on YPD plates containing different concentrations of sorbitol or ethanol. Sorbitol was used to generate high osmotic stress. The growth was examined after 2 days for YPD control medium, or 6 to 10 days for plates containing sorbitol or ethanol.

Tolerance to HMF, furfural and acetate was performed in 1 ml liquid synthetic medium containing the individual inhibitors in a 24-well plate. The same pre-culture used for the spot assay was inoculated into medium containing a range of concentrations of each inhibitor at an initial OD_630_ of 0.2. The OD was measured after 48 to 72 h using Synergy H1 Hybrid Reader (BioTek, Winooski, VT, USA). Each experiment was performed in duplicate with independent cultures.

### Origin and preparation of lignocellulose hydrolysates

Three different pretreated lignocellulosic biomass materials (*Arundo donax*, spruce tree and a 50/50 mixture of wheat straw and hay) were used to evaluate the fermentation performance of the final strain. They were obtained from Chemtex (Tortona AL, Italy), SEKAB E-Technology AB (Örnsköldsvik, Sweden), and KaHo Sint-Lieven (Ghent, Belgium), respectively. Pretreatment of *Arundo donax* and spruce were performed by pure steam explosion and SO_2_ impregnated steam explosion, respectively. The wheat straw/hay mixture was pretreated using 0.4 M NaOH at 25°C for 24 h. The residue was washed 3 times with RO water. The *Arundo donax* and spruce pretreated materials were hydrolyzed at an initial pH of 4.8, using enzyme complex ACCELLERASE® 1500 for 48 h at 53°C according to the protocol from the manufacturer. Enzyme hydrolysis of pretreated wheat straw/hay mixture was done using Novozymes cellulase complex (NS50013) and beta-glucosidase (NS50010) at 50°C, pH 4.5 for 24 h. Fermentation was done at a solid loading of 12% (w/v) for spruce and *Arundo donax* hydrolysate and 19% (w/v) for wheat straw/hay hydrolysate. For selection of D-xylose growing and inhibitor tolerant strains after the genome shuffling step, pretreated spruce material was used before enzymatic hydrolysis.

### Simultaneous Saccharification and Fermentation (SSF)

All SSF experiments were carried out in 2.5 L bioreactors (Biostat A, B. Braun Biotech International, Melsungen, Germany and Biostat A plus, Sartorius, Melsungen, Germany) with a final working weight of 1.2 kg. The experiments were carried out with an initial water insoluble solids (WIS) content of 10% according to [45]. The same batches of pretreated spruce and arundo were used as in [45]. The full composition of the pretreated material is given in that reference. To obtain the desired WIS content, the pretreatment slurry was diluted with sterile deionized water. The pH was maintained at 5.0 throughout the fermentation by automatic addition of 3 M NaOH and the stirring speed was kept at 500 rpm. Celluclast 1.5 L, and Novozym 188, a β-glucosidase provided by Novozymes A/S (Bagsvaerd, Denmark) were used and the enzyme dosage for SSF experiments was 10 mg/g WIS for Celluclast 1.5 L and 500 nkat/g DW for Novozym 188. The SSF medium was supplemented with 0.5 g/L (NH_4_)_2_HPO_4_, 0.025 g/L MgSO_4_ · 7H_2_O and 1.0 g/L yeast extract. An initial yeast concentration of 4 g DW/L was used. All SSF experiments were performed in duplicate. The initial D-xylose content in GS1.11-26 experiments on Arundo was higher than in the corresponding previous experiments with Ethanol Red [45]. The reason was that the time for autoclaving the slurry was increased from 20 min to 1 h prior to SSF to prevent contamination, which caused hydrolysis of xylo-oligomers.

The yeast to be used in SSF was obtained by propagation from solid YPD medium into aerobic batch cultivation on glucose, followed by aerobic fed-batch cultivation on arundo or spruce liquid fractions. A defined medium with glucose 20 g/L as the carbon source [44] was used for the batch phase. However, no uracil was added in the present case. The volume in the bioreactor was 0.7 L and pH was maintained at 5.0 throughout the cultivation by automatic addition of 3 M NaOH. The cultivation was initiated by adding 20.0 mL of inoculum (prepared according to [44]) to the bioreactor. Aeration was maintained at 1.2 L/min and the stirrer speed was kept at 800 rpm. The oxygen and CO_2_ content in the exhaust gas were measured with a gas analyzer (1313 Fast Response Triple-gas Monitor, INNOVA, Denmark). Upon depletion of the ethanol produced in the batch phase, the feeding of liquid fraction from spruce or Arundo was initiated. A total of 1.0 L of autoclaved liquid fraction was used for feeding. In case of arundo, 40 g of glucose was added to 1.0 L of liquid fraction prior to autoclaving. The feeding followed a linear profile with an initial feed rate of 0.04 L/h which was increased linearly to 0.10 L/h during 16 h of cultivation according to [79]. The aeration during the fed-batch phase was maintained at 1.4 L/min and the stirrer speed was kept at 800 rpm. After cultivation, the cells were harvested by centrifugation in 700 mL flasks for 8 min at 3000 rpm using a HERMLE Z 513 K centrifuge (HERMLE Labortechnik, Wehingen, Germany). The pellets were resuspended in 0.9% NaCl solution in order to obtain a cell suspension with a cell mass concentration of 75 g DW/L.

### Analysis of substrates and metabolites

The metabolites and substrate content were analysed using high performance liquid chromatography (HPLC). In the case of hydrolysates, samples were centrifuged in 2 mL microcentrifuge tubes at 14,000 rpm for 5 min (Z 160 M, HERMLE Labortechnik, Wehingen, Germany). The supernatant was filtered using 0.2 μm filters, and the filtered samples were stored at −20°C. The sugar concentrations were determined using a polymer column (Aminex HPX87P, BioRad Laboratories, München, Germany) at 85°C. MilliQ water was used as eluent, with a flow rate of 0.6 ml/min. Ethanol, glycerol, acetate, HMF and furfural were analyzed using an Aminex HPX87H column (BioRad Laboratories, München, Germany) at 60°C. The eluent was 5 mM H_2_SO_4_ with a flow rate of 0.6 ml/min. The compounds of interest were detected with a refractive index detector (Waters 2410, Waters, Milford, MA, USA) or with a UV detector at a wavelength of 210 nm (Waters 2487, Waters, Milford, MA, USA). Metabolites and substrates in fermentation experiments with synthetic or complex medium were analyzed by Waters Isocratic Breeze HPLC system using ion-exchange column WAT010290 and a refractive index detection system (Waters 2414 RI detector). Column temperature was maintained at 75°C and 5 mM H_2_SO_4_ was used as eluent at a flow rate of 1 ml/min.

### Cell mass concentration

Optical Density (OD_600nm_) was used to estimate cell dry weight (DW). The DW for inocula was measured by filtering a 10 ml culture aliquot in pre-weighed 0.2 mm Supor Membrane disc filters (PALL Corporation, USA), washing the filter with MilliQ water, and drying it in a microwave oven at about 150 watt for 15–20 min to constant weight. The correlation between dry weight (DW) and OD_600_ was measured for each strain tested.

### Calculations

Ethanol yield in SSF was calculated based on the total amount of fermentable sugars, which includes glucose, mannose, and galactose, present in the pretreatment slurry, including monomers, oligomers, and polymers (glucan fibers). In SHF, the total amount of fermentable sugar monomers was taken into account. The specific D-xylose consumption rate was calculated according to the standard protocol described previously [1]. The biomass of the initial inoculum was used for calculation of sugar consumption in the fermentation experiments with lignocellulose hydrolysates, since the biomass cannot be accurately measured during the actual fermentation process.

## Abbreviations

DW: dry weight; OD: optical density; YPD: Yeast extract peptone dextrose; YPX: Yeast extract peptone D-xylose; SC: synthetic complete medium; HPLC: high performance liquid chromatography; XI: D-xylose isomerase; XR: D-xylose reductase; XDH: xylitol dehydrogenase; XK: xylulokinase; VHG: very high gravity; LB: Luria-Bertani; PCR: polymerase chain reaction; SSF: Simultaneous saccharification and fermentation; SHF: Separate hydrolysis and fermentation.

## Competing interests

EB declares competing financial interests. Goethe-University Frankfurt has filed a patent application concerning the use of the *C. phytofermentans* xylose isomerase. EB is named as an inventor. The patent application has been sold to Butalco GmbH (Switzerland). EB is co-founder and shareholder of Butalco GmbH. The other authors declare that they have no competing interests.

## Authors’ contributions

MD, HD, EB and JT designed the experiments. MD, HD, YL, SM, SD, TDA and BB performed the experiments. MF, FD, AV and GL provided academic supervision and helped in the experimental design and data analysis. MD, HD, GL, EB and JT analyzed the results and wrote the manuscript. All authors read and approved the final manuscript.
